# [HgX_2_] Linear Group Enabled Ultraviolet Birefringent Crystal RbHg_5_Br_11_ with Strong Optical Anisotropy and Wide Bandgap

**DOI:** 10.1002/advs.202514304

**Published:** 2025-09-09

**Authors:** Yinxia Du, Wangfei Che, Yunfei Shi, Juanjuan Lu, Xiaodong Zhou, Ting Liu, Shilie Pan, Junjie Li

**Affiliations:** ^1^ State Key Laboratory of Chemistry and Utilization of Carbon‐Based Energy Resources College of Chemistry Xinjiang University Urumqi 830017 China; ^2^ Research Center for Crystal Materials State Key Laboratory of Functional Materials and Devices for Special Environmental Conditions Xinjiang Key Laboratory of Functional Crystal Materials Xinjiang Technical Institute of Physics & Chemistry Chinese Academy of Sciences 40–1 South Beijing Road Urumqi 830011 China; ^3^ Center of Materials Science and Optoelectronics Engineering University of Chinese Academy of Sciences Beijing 100049 China

**Keywords:** [HgX_2_], birefringent crystals, Hg‐halides, linear units, optical anisotropy

## Abstract

Birefringent crystals are pivotal for modern optical modulation technologies, yet developing high‐performance birefringent materials with large birefringence (Δ*n*), wide bandgaps, and scalable synthesis remains a significant challenge. Different from the traditional planar [MQ_3_] and distorted [MQ_n_] (n ≥ 4) polyhedral units, a “linear‐group” design strategy is proposed, targeting heavy‐metal halides with [HgX_2_] (X = halides) coordination modes to exploit their inherent polarizability anisotropy. Through systematic experimental investigations in the ternary A‐Hg‐X (A = Rb, Cs; X = Br, I) system, six novel Hg‐based halides were synthesized. Notably, RbHg_5_Br_11_ with linear [HgBr_2_] units demonstrates excellent optical properties, including a wide bandgap (3.73 eV) and large Δ*n_(__cal.__)_* (0.35@546 nm). Importantly, the compound displays a good crystal growth habit, and the high‐quality RbHg_5_Br_11_ single crystal can be grown by the simple solution method. Theoretical calculations reveal that the strong optical anisotropy arises from the aligned [HgBr_2_] linear units. The results demonstrate that RbHg_5_Br_11_ is a promising birefringent material and give some new insights for designing high‐performance optical materials based on the linear units with high polarizability anisotropy.

## Graphical abstract

Based on the “linear‐group” design strategy, a high performance ultraviolet birefringent material RbHg_5_Br_11_ with aligned linear [HgBr_2_] units, demonstrating a large birefringence (Δ*n* ≈ 0.35@546 nm); a wide optical bandgap (≈3.73 eV); as well as a good crystal growth habit; is rationally designed; and fabricated by a simple solution method.

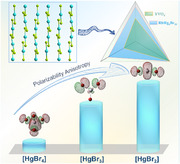

## 1 Introduction

Birefringent materials play a crucial role in light manipulation for preparing optical isolators, polarizers, and phase retarders.^[^ ^1^, ^2^, ^3^ ^]^ These components are widely used in numerous scientific and engineering fields, including optical communication, polarimetry, and laser technology.^[^ ^4^, ^5^ ^]^ Usually, the performance of devices is principally governed by the phase retardance (*φ*) between ordinary and extraordinary rays—a parameter determined by the material's birefringence (∆*n*) and the device thickness (*d*).^[^ ^6^, ^7^, ^8^, ^9^ ^]^ In the ultraviolet (UV) and visible spectral regions, metal oxides and halides have been demonstrated as the alternative systems, and YVO_4_, *α*‐BaB_2_O_4_, and MgF_2_ have been commercially applied.^[^ ^10^, ^11^, ^12^, ^13^, ^14^, ^15^, ^16^ ^]^ However, owing to the intrinsic drawbacks like small Δ*n* in MgF_2_ (0.012@546 nm) and *α*‐BaB_2_O_4_ (0.122@546 nm), not‐short‐enough UV cut‐off edge (400 nm) in YVO_4_, their applications are limited in current optical techniques, especially for the compact high‐efficiency UV optical equipment.^[^ ^17^, ^18^, ^19^, ^20^ ^]^ Therefore, developing new birefringent materials with large Δ*n*, wide bandgaps, and good crystal growth habits is highly expected.^[^ ^21^, ^22^ ^]^

From the structure‐property relationship perspective, searching for fundamental building blocks (FBBs) with high polarizability anisotropy is paramount.^[^ ^23^, ^24^, ^25^, ^26^, ^27^, ^28^, ^29^ ^]^ Over the past decades, numerous attempts have been made, leading to the development of various new birefringent candidates,^[^ ^30^, ^31^ ^]^ such as CaCO_3_, *α*‐BaB_2_O_4_, TiO_2_, HgBr_2_, *α*‐SnF_2_ and Bi_12_SiO_20_, based on the *π*‐conjugated planar units ([CO_3_]^2−^, [NO_3_]^−^ and [BO_3_]^3−^),^[^ ^32^ ^]^ distorted metal polyhedra ([TiS_6_]^8−^, [HgCl_4_]^2−^ and [NbO_6_]^7−^) and stereochemical activated cations (Sn^2+^, Pb^2+^ and Bi^3+^).^[^ ^33^, ^34^, ^35^ ^]^ In contrast to the planar [MQ_3_] (M = C, B, N, Hg…; Q = O, F, S) and polyhedral [MQ_n_] (M = B, Hg, Pb, Si…; Q = O, F, S; n = 4, 5, 6) units, the emerging linear [MQ_2_] (M = B, Hg, I; Q = O, S, halides) units exhibit high polarizability anisotropy,^[^ ^36^, ^37^, ^38^, ^39^ ^]^ showing promising applications in the design of advanced birefringent materials.^[^ ^40^, ^41^, ^42^, ^43^, ^44^ ^]^ However, the low formation probability of these linear units makes designing [MQ_2_] based birefringent materials challenging.^[^ ^45^, ^46^ ^]^

**Figure 1:**
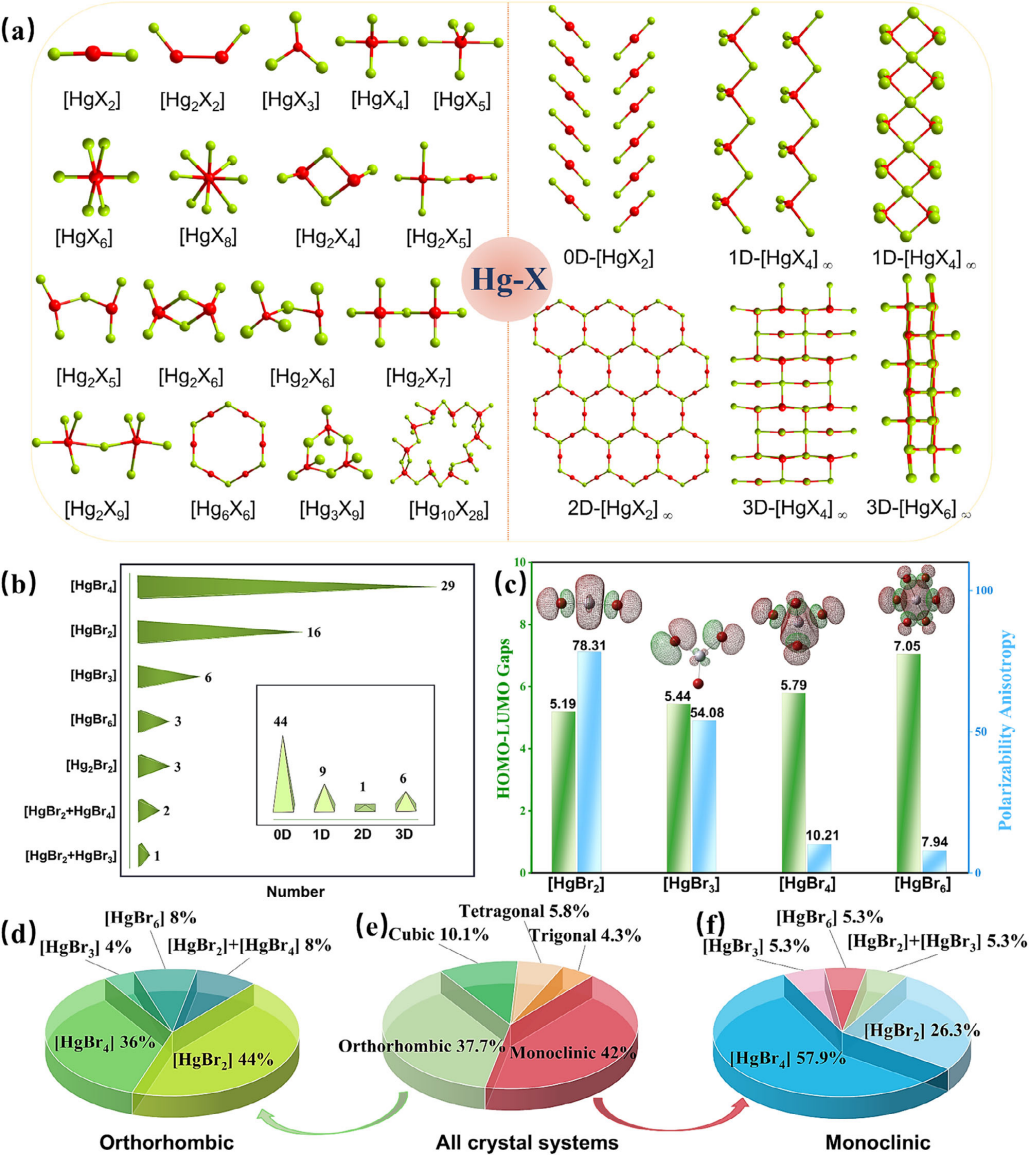
a) [Hg_m_X_n_] (m, n = positive integer) units and their connection modes. b) Statistical investigation on the structures of Hg‐based bromides in the ICSD. Insert showing the dimension distribution of the formed [Hg_m_Br_n_] (m = 1, 2; n = 2, 3, 4, and 6) units in the compounds. c) The calculated polarizability anisotropy and HOMO‐LUMO gaps of the [Hg_m_Br_n_] units. d–f) The chemical and structural diversities of Hg‐based compounds.

Based on a systematic investigation in the Inorganic Crystal Structure Database (ICSD version 5.4.0 (build 20250403–0947)), the [HgX_2_] (X = Cl, Br, and I) shows a relatively high formation probability (≈9.2%) in the reported Hg‐based halides, as shown in **Figure** **1**a,b. Considering the large polarizability anisotropy in the linear [HgX_2_] and [Hg_2_X_2_] units (Figure 1c), as well as the abundant structural and chemical diversities (Figure 1d–f), and good crystal growth habits,^[^ ^45^, ^46^, ^47^ ^]^ the Hg‐based halide systems were re‐examined by the simple solution method and solid‐state reaction in this work.^[^ ^48^, ^49^ ^]^ Six new compounds, AHg_5_Br_11_ (A = Rb, Cs), RbHg_2_Br_5_, Rb_7_Hg_3_Br_13_, Rb_3_Hg_2_Br_7_ and Rb_3_Hg_2_I_7_, were rationally designed and fabricated in experiment. Among them, AHg_5_Br_11_ (A = Rb, Cs) crystallize in the monoclinic *C*2/*m* space group, and are composed of linear [HgBr_2_] and polyhedral [RbBr_10_] units. RbHg_5_Br_11_ displays a wide bandgap (3.73 eV). Meanwhile, it possesses the largest reported ∆*n* (0.35@546 nm, 29.1 times that of commercial MgF_2_ crystal) among the ternary Hg‐halides (Table S1, Supporting Information). Experimental and theoretical results uncover that the strong optical anisotropy in RbHg_5_Br_11_ originates from the directionally arranged [HgBr_2_] linear units. Moreover, a 5 × 3 × 1 mm^3^ RbHg_5_Br_11_ single crystal was achieved by the simple solution method, demonstrating a good crystal growth habit, which is important for the practical applications of an emerging ∆*n* candidate.

## 2 Results and Discussion

The single crystals were picked out for crystal structural determination under an optical microscope. The detailed information on the chemical syntheses and structural characterizations can be seen in the Experimental section. The results of single crystal X‐ray diffraction (XRD) show that AHg_5_Br_11_ crystallizes in the monoclinic *C*2/*m* space group (No. 12), RbHg_2_Br_5_, Rb_3_Hg_2_Br_7,_ and Rb_3_Hg_2_I_7_ in *P*2_1_/*c* (No. 14), while Rb_7_Hg_3_Br_13_ in the orthorhombic *Pnma* space group (No. 62) (**Table** **1**). The detailed crystallographic data and refined parameters of the six compounds, including atomic coordinates, equivalent isotropic displacement parameters, selected bond lengths and angles, are given in Tables S2–S25 (Supporting Information), respectively. To further identify the chemical compositions of the six new compounds, the energy‐dispersive X‐ray spectroscopy (EDS) spectra and element mappings were tested, demonstrating the presence of Cs/Rb, Hg, Br/I in the target compounds (Figure S1, Supporting Information).

**Table 1:** The investigation of six selected ternary halides.

Compounds	RbHg_5_Br_11_	CsHg_5_Br_11_	RbHg_2_Br_5_
Formula weight [g·mol^−1^]	1967.43	2014.78	886.20
Crystal system	Monoclinic	Monoclinic	Monoclinic
Space group	*C*2/*m*	*C*2/*m*	*P*2_1_/*c*
*a* (Å)	12.2892(9)	12.358(3)	8.4203(7)
*b* (Å)	15.1269(10)	15.200(3)	10.3866(10)
*c* (Å)	6.8202(5)	6.9384(15)	13.1634(9)
*β/°*	119.004(3)	119.750(8)	107.994(3)
*V* [Å^3^]	1108.85(14)	1131.5(4)	1094.62(16)
*Z*	2	2	4
*ρ*_calc_ [g·mol^−3^]	5.893	5.914	5.377
*µ* [mm^−1^]	56.473	54.789	50.611
*F* (000)	1644.0	1680.0	1488.0
Completeness [%]	99.7	97.8	98.9
Data/restraints/parameters	1283/0/47	1311/0/47	2483/0/74
2*Θ* range for data collection/°	4.642 to 50.698	4.648 to 54.964	5.096 to 54.986
GOF on *F* ^2^	1.168	0.912	1.051
Final *R* indexes [*I*>=2*σ*(*I*)]^a)^	0.0367, 0.0769	0.0461, 0.1100	0.0423, 0.0870
Final *R* indexes [all data]^a)^	0.0379, 0.0776	0.0626, 0.1230	0.0755, 0.1001
Largest diff. peak/hole/[e·Å^−3^]	2.03/−2.71	1.78/−1.30	1.82/−1.38
Compounds	Rb_3_Hg_2_Br_7_	Rb_3_Hg_2_I_7_	Rb_7_Hg_3_Br_13_
Formula weight [g·mol^−3^]	1216.96	1545.89	2238.89
Crystal system	Monoclinic	Monoclinic	Orthorhombic
Space group	*P*2_1_/*c*	*P*2_1_/*c*	*Pnma*
*a* (Å)	11.9907(10)	12.7126(7)	13.4808(17)
*b* (Å)	7.3301(6)	7.7407(3)	26.463(3)
*c* (Å)	20.2864(13)	21.9061(12)	9.4581(12)
*β/°*	101.302(2)	101.349(2)	90
*V* (Å^3^)	1748.5(2)	2113.51(18)	3374.1(7)
*Z*	4	4	4
*ρ*_calc_ [g·mol^−1^]	4.623	4.858	4.407
*µ* [mm^−1^]	41.797	31.570	39.038
*F* (000)	2064.0	2568.0	3816.0
Completeness [%]	98.2	99.7	99.9
Data/restraints/parameters	3981/0/109	4850/0/111	3520/0/112
2*Θ* range for data collection/°	3.464 to 55.038	3.792 to 55.074	4.574 to 52.726
GOF on *F* ^2^	1.098	1.022	1.056
Final *R* indexes [*I*>=2*σ*(*I*)]^a)^	0.0415, 0.1049	0.0350, 0.0633	0.0231, 0.0457
Final *R* indexes [all data]^a)^	0.0513, 0.1119	0.0585, 0.0732	0.0292, 0.0476
Largest diff. peak/hole/[e·Å^−3^]	2.36/−1.588	2.68/−2.72	1.31/−0.92

^a)^ *R*_1_ = Σ||*F*_o_| – |*F*_c_||/Σ|*F*_o_| and *w* *R*_2_ = [Σ*w*(*F*_o_^2^ – *F*_c_^2^)^2^/Σ*w* *F*_o_^4^]^1/2^ for *F*_o_^2^ >2*σ*(*F*_o_^2^).

**Figure 2:**
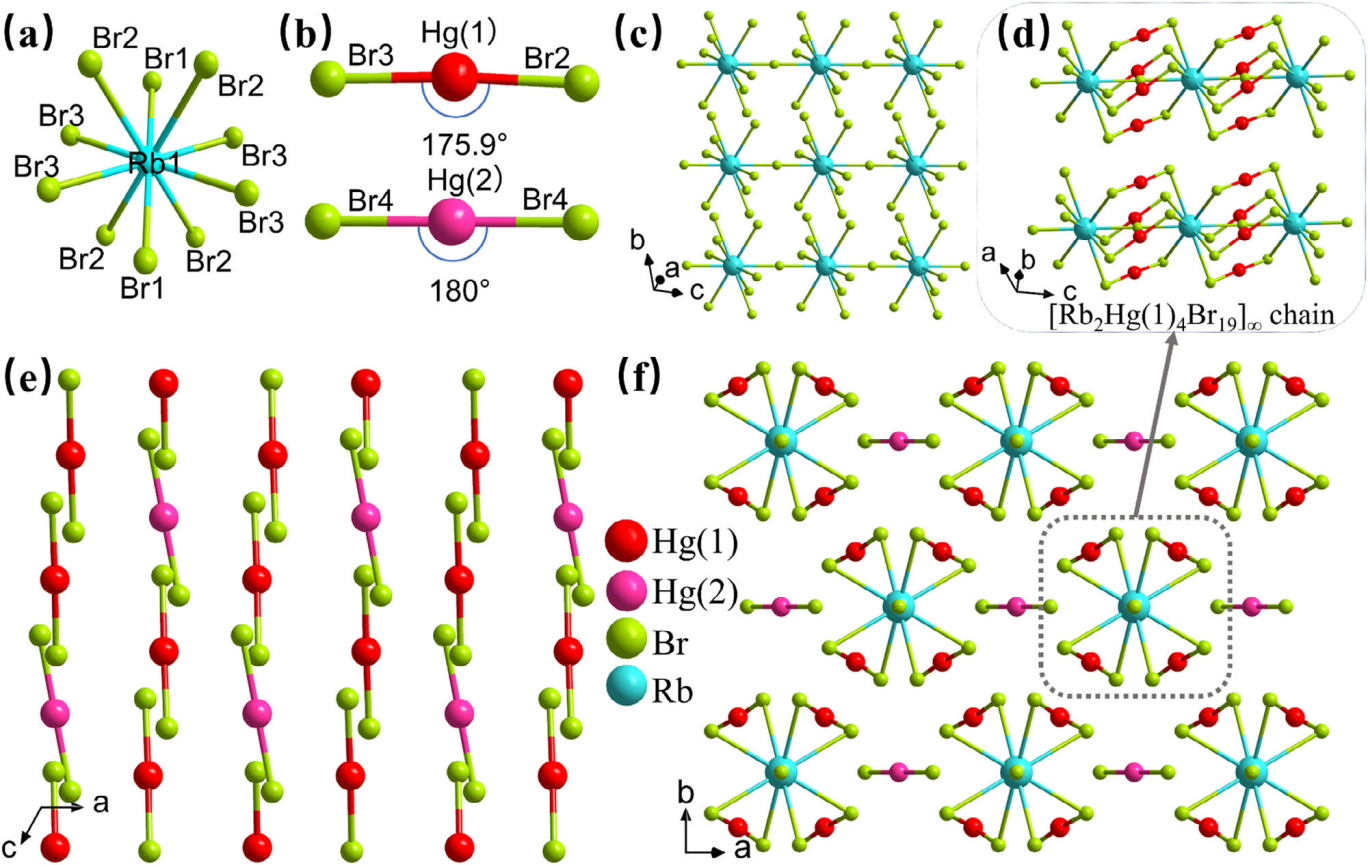
Crystal structure of RbHg_5_Br_11_. a,b) The coordination modes of Rb and Hg. c) The formed [Rb_2_Br_19_]_∞_ 1D chain. d) The branched 1D [Rb_2_Hg(1)_4_Br_19_]_∞_ chain structure. e) The arrangement of isolated [Hg(1)Br_2_] and [Hg(2)Br_2_] units. f) The resulting 3D crystal structure.

Based on the coordination modes of Hg atom, the six compounds can be divided into three categories: i) containing linear [HgBr_2_] units in AHg_5_Br_11_ (A = Rb, Cs); ii) planar [Hg_2_Br_5_] units in RbHg_2_Br_5_; iii) tetrahedral [HgX_4_] (X = Br, I) units in Rb_7_Hg_3_Br_13_, Rb_3_Hg_2_Br_7_ and Rb_3_Hg_2_I_7_. Since RbHg_5_Br_11_ and CsHg_5_Br_11_ are isostructural compounds, RbHg_5_Br_11_ is utilized as an example to illustrate their crystal structures herein (**Figure** **2**; Figure S2, Supporting Information). RbHg_5_Br_11_ crystallizes in the centrosymmetric (CS) *C*2/*m* (No. 12) space group with cell parameters *a*  =  12.2892(9) Å, *b*  =  15.1269(10) Å, *c*  =  6.8202(5) Å, *β* = 119.004(3)°, and *Z* = 2 (Table 1). In its asymmetric unit, there are two independent crystallographic Hg, one Rb, and four Br atoms. The Rb, Hg(1) and Hg(2) atoms are located at the Wyckoff positions of 2*d*, 8*j* and 2*c*, respectively, and Br(1–4) atoms at 2*b*, 8*j*, 8*j* and 4*i* positions. Each Rb atom is linked with ten Br atoms to form a [RbBr_10_] polyhedral unit (Figure 2a). Each Hg atom bonds two Br atoms with Hg─Br bond length *d*_Hg‐Br_ = 2.4192(13)‐2.4312(10) Å, forming two types of linear [HgBr_2_] units distinguished by their bond angles: ∠Br‐Hg(1)‐Br = 175.92(4)° and ∠Br‐Hg(2)‐Br = 180.00°, as shown in Figure 2b. To further demonstrate the chemical bonding, the Raman spectrum of RbHg_5_Br_11_ was tested on a LABRAM HR Evolution spectrometer. As shown in Figure S3 (Supporting Information), the peak at 179 cm^−1^ could be attributed to the characteristic vibrations of Hg─Br bonds,^[^ ^50^ ^]^ and the peaks at 50 and 70 cm^−1^ are related to the Rb─Br bonds in the compound.^[^ ^51^ ^]^ The formed [RbBr_10_] polyhedral units are connected by corner‐sharing to build the [Rb_2_Br_17_] 1D infinite chains (Figure 2c), which are further bonded with the [Hg(1)Br_2_] to form a branched 1D [Rb_2_Hg(1)_4_Br_19_]_∞_ chain structure (Figure 2d). Both of the linear [HgBr_2_] units are isolated with each other in the structure (Figure 2e). The [Hg(2)Br_2_] units are located at the gaps of [Rb_2_Hg(1)_4_Br_19_]_∞_ chain structures, resulting in the final 3D crystal structure of RbHg_5_Br_11_ (Figure 2f). In contrast to AHg_5_Br_11_ (A = Rb, Cs), which consist of linear [HgBr_2_] and polyhedral [ABr_10_] (A = Rb, Cs) units, RbHg_2_Br_5_ features planar [Hg_2_Br_5_] dimers and [RbBr_9_] polyhedra (Figure S4, Supporting Information). The remaining compounds Rb_7_Hg_3_Br_13_, Rb_3_Hg_2_Br_7,_ and Rb_3_Hg_2_I_7_ contain either [HgX_4_] (X = Br or I) or [Hg_2_Br_7_] dimers, along with Rb‐X polyhedral groups (Figures S5–S7, Supporting Information). Moreover, based on the statistical analysis, RbHg_5_Br_11_, RbHg_2_Br_5_, Rb_7_Hg_3_Br_13,_ and Rb_3_Hg_2_Br_7_ are the first reported compounds in the Rb‐Hg‐Br ternary phase diagram (Figure S8, Supporting Information).

**Figure 3:**
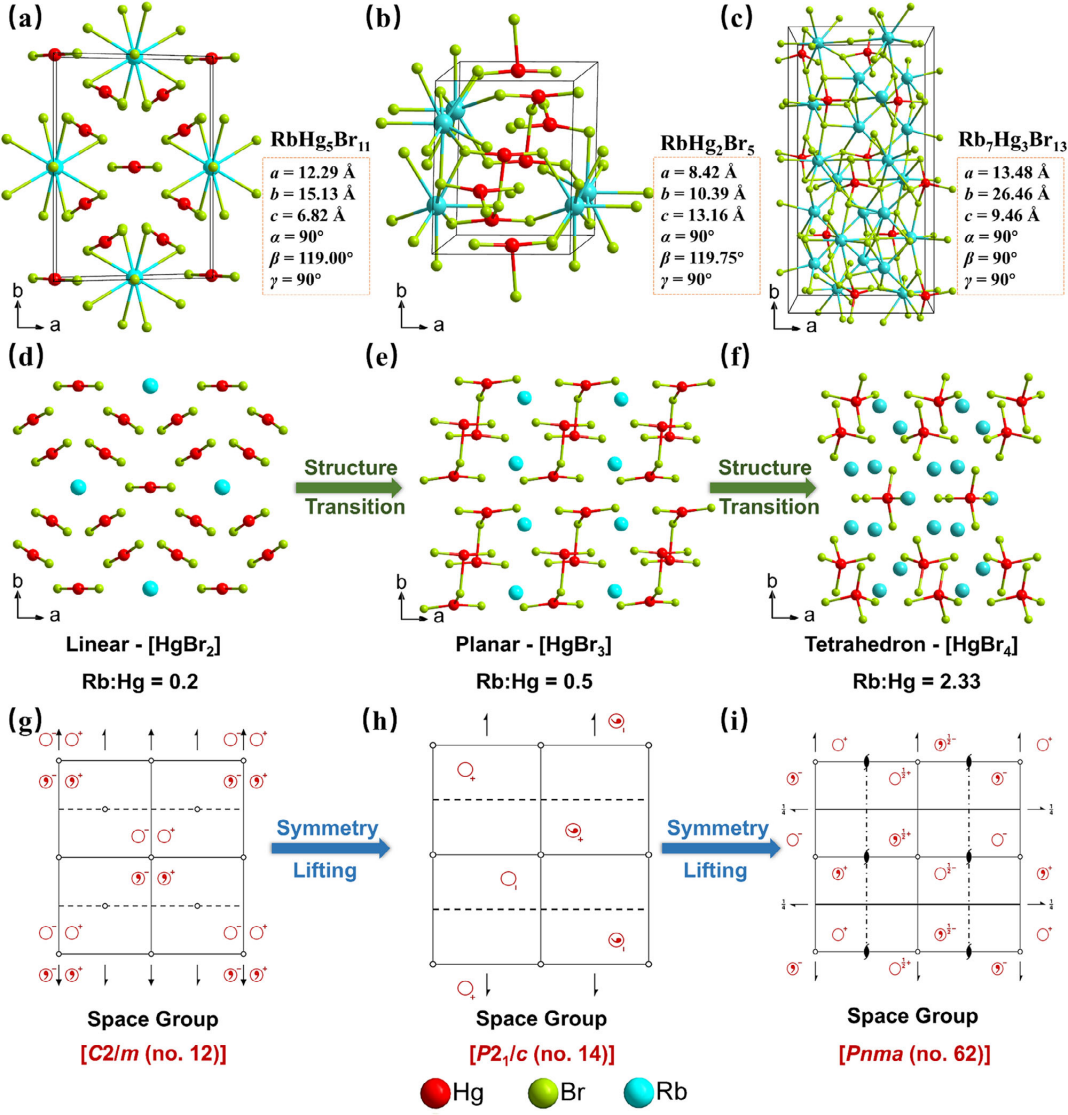
a–f) Structural comparison between RbHg_5_Br_11_, RbHg_2_Br_5,_ and Rb_7_Hg_3_Br_13_. g–i) Symmetry changes from *C*2/*m* (RbHg_5_Br_11_) to *P*2_1_/*c* (RbHg_2_Br_5_), *Pnma* (Rb_7_Hg_3_Br_13_).

To illustrate the correlations between Rb/Hg ratio and crystallographic symmetry, a detailed structural analysis on RbHg_5_Br_11_ (*C*2/*m*), RbHg_2_Br_5_ (*P*2_1_/*c*), and Rb_7_Hg_3_Br_13_ (*Pnma*) was carried out. As illustrated in **Figure** **3**a–c, the unit cell parameters (*a*, *b*, *c*, *α*, *β*, *γ*) and *b‐*axis projections demonstrate that all three compounds adopt 3D framework structure composed of isolated [HgBr_n_] (n = 2–4) and charge‐balancing [RbBr_n_] (n = 6–10) units. Notably, with the increase of Rb^+^ content from RbHg_5_Br_11_ (Rb/Hg = 0.2) to RbHg_2_Br_5_ (Rb/Hg = 0.5) and Rb_7_Hg_3_Br_13_ (Rb/Hg = 2.33), the isolated [HgBr_n_] units in above three structures evolve from linear [HgBr_2_] (in RbHg_5_Br_11_) to planar [HgBr_3_] (in RbHg_2_Br_5_) and finally tetrahedral [HgBr_4_] (in Rb_7_Hg_3_Br_13_) (Figure 3d–f). Correspondingly, the Rb/Hg ratio variation drives a concomitant symmetry enhancement, as depicted in Figure 3g–i. The space group transitions from low symmetry *C*2/*m* (No. 12) in RbHg_5_Br_11_ to higher symmetries *P*2_1_/*c* (No. 14) in RbHg_2_Br_5_ and *Pnma* (No. 62) in Rb_7_Hg_3_Br_13_. The results indicate that the targeted Rb^+^ incorporation can modulate structural symmetry while preserving isolated [HgBr_n_] units in the Hg‐based halide systems.

**Figure 4:**
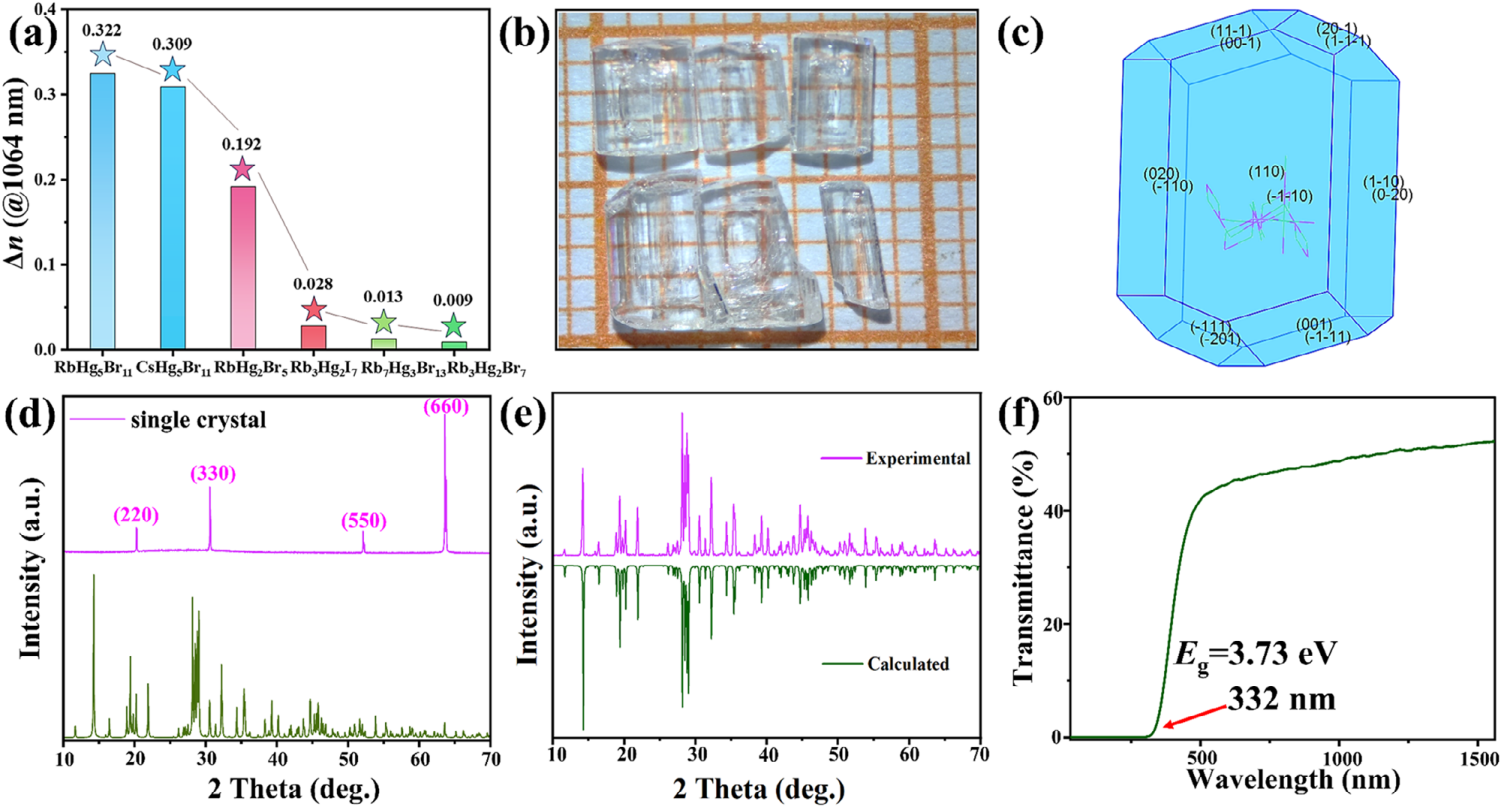
a) The calculated ∆*n* of the six compounds at 1064 nm. b,c) The optical image b) and the theoretical morphology c) of RbHg_5_Br_11_; d,e) The XRD patterns of single crystal d) and polycrystalline powder samples e) of RbHg_5_Br_11_; f) UV–vis‐NIR transmittance spectrum of RbHg_5_Br_11_.

Considering the linear unit has a high polarizability anisotropy: *δ*[HgBr_2_] > *δ*[HgBr_3_] > *δ*[HgBr_4_] (Figure 1c), the refractive indices along the optical axes (*n*_x_, *n*_y_, *n*_z_) direction of the six compounds were investigated by the density functional theory (DFT) calculations.^[^ ^52^, ^53^, ^54^, ^55^, ^56^ ^]^ Among them, RbHg_5_Br_11_ exhibits a strong optical anisotropy with a ∆*n* of 0.32, larger than the ones of 0.31 in CsHg_5_Br_11_, 0.19 in RbHg_2_Br_5_, 0.013 in Rb_7_Hg_3_Br_13_, 0.009 in Rb_3_Hg_2_Br_7_, 0.028 in Rb_3_Hg_2_I_7_ at 1064 nm, respectively (**Figure** **4**a; Figure S9, Supporting Information), while smaller than the recently reported 0.994@546 nm in CsICl_2_ due to the strong polarizability anisotropy in [ICl_2_] linear units and their parallel alignment in the structure.^[^ ^8^ ^]^ Moreover, the birefringence difference between RbHg_5_Br_11_ and CsHg_5_Br_11_ could be related to the spacing fluctuations between the [A_2_Hg(1)_4_Br_19_]_∞_ (A = Rb and Cs) chains induced by the Rb/Cs substitutions, similar to the case in AMgGeSe_3_ (A = Li and Na).^[^ ^5^ ^]^ To test the optical properties of RbHg_5_Br_11_, the millimeter‐sized single crystals were grown by the solution method (Figure 4b,c). The single crystal and powder XRD results confirm the obtained high quality RbHg_5_Br_11_ single crystals, and it shows a preferential growth of {110} plane (Figure 4c–e). To detect the optical bandgap and UV transparency range, the UV–vis–NIR transmittance spectrum of RbHg_5_Br_11_ crystal was tested on a Shimadzu SolidSpec‐3700DUV spectrophotometer. It shows that the RbHg_5_Br_11_ crystal has a shorter UV cut‐off edge (332 nm) than the commercial YVO_4_ and TiO_2_ (400 nm) birefringent crystals.^[^ ^57^, ^58^ ^]^ The corresponding experimental bandgap was determined to be 3.73 eV, matched with the result of the UV–vis‐NIR diffuse reflectance spectrum (Figure S10a, Supporting Information). It is larger than the one of 3.37 eV in CsICl_2_ with [ICl_2_] linear units, due to the wider HOMO‐LUMO gap in [HgBr_2_] units compared to the linear [ICl_2_] units.^[^ ^8^ ^]^ Correspondingly, the optical bandgaps of Rb_7_Hg_3_Br_13_, Rb_3_Hg_2_Br_7,_ and Rb_3_Hg_2_I_7_ were measured to be 3.45, 3.38, and 2.67 eV respectively (Figure S10b–d, Supporting Information).

**Figure 5:**
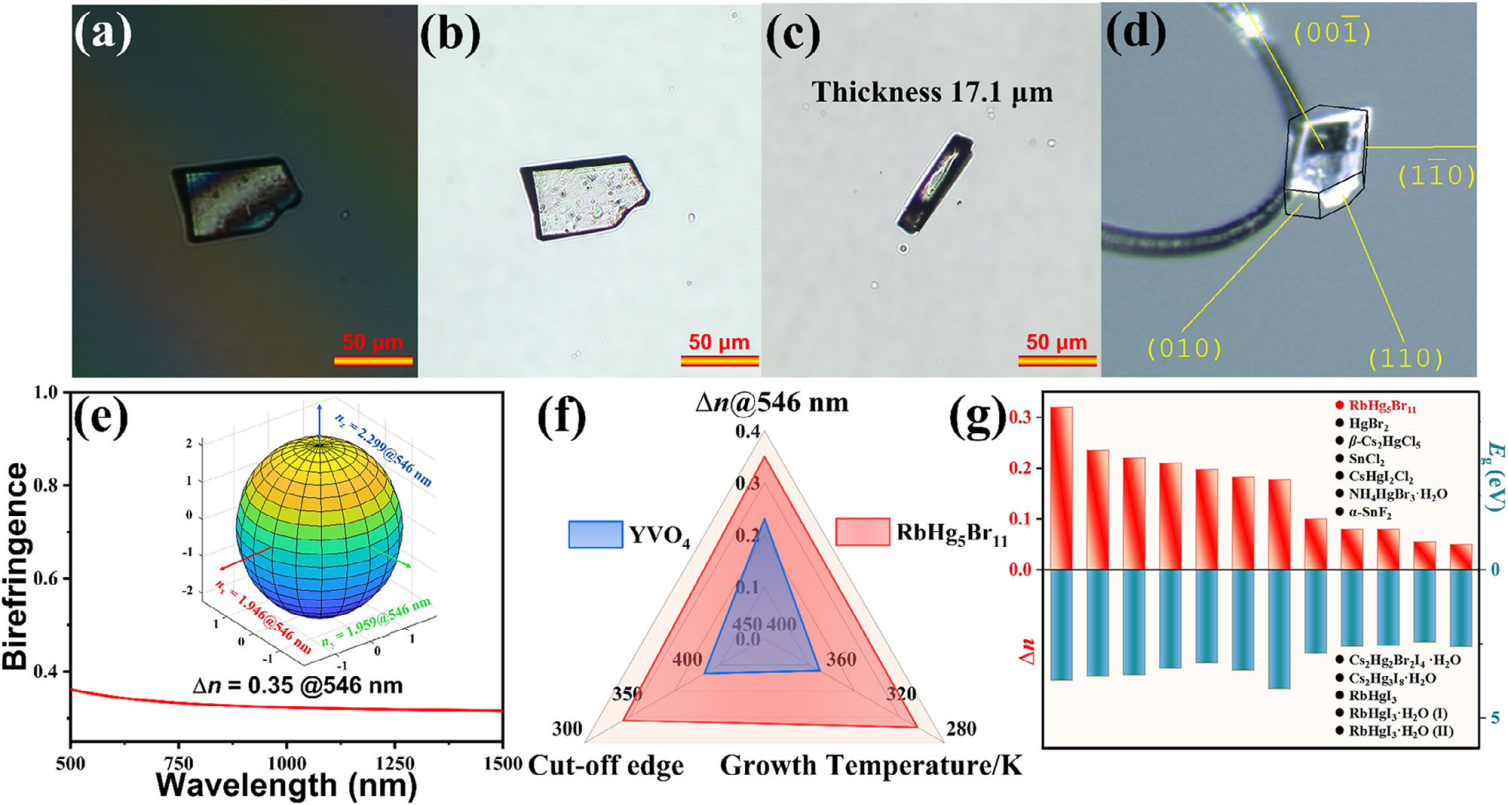
a) The rotation of compensatory; b) Photograph of RbHg_5_Br_11_ crystal; c) The thickness of RbHg_5_Br_11_ for the RID measurement; d) The crystal orientation of RbHg_5_Br_11_ indexed by single‐crystal XRD; e) Calculated birefringence of RbHg_5_Br_11_ (Inset showing the triaxial ellipsoid of three refractive indices along crystallographic axes); f) The property comparison between RbHg_5_Br_11_ and benchmark YVO_4_; g) Statistical analysis of ∆*n* and bandgap in typical birefringent candidates.

To evaluate the optical anisotropy of the RbHg_5_Br_11_ single crystal, the refractive index difference (RID) of the compound was tested using a Carl Zeiss Axioscope 5 polarizing microscope. As shown in **Figure** **5**a–d, the difference in the optical path (*D*) and sample thickness was determined to 4433.3 nm, 17.1 µm under the irradiation of 546 nm. The RID subsequently was computed as 0.259@546 nm, smaller than the calculated ∆*n* of 0.35@546 nm in Figure 5e, owing to a deviation caused by misalignment between the normal direction of (00$\bar{1}$) plane and the optical principal axes. Moreover, the RIDs of CsHg_5_Br_11_ and RbHg_2_Br_5_ were measured to be 0.198 and 0.114 at 546 nm (Figure S11, Supporting Information), respectively. To the best of our knowledge, RbHg_5_Br_11_ shows the largest ∆*n* among the reported ternary Hg‐halides (Figure 5g), and it achieves a good balance between large ∆*n* (0.35, larger than the ones of 0.012 in MgF_2_, 0.204 in YVO_4_, 0.122 in *α*‐BaB_2_O_4_ at 546 nm) and short UV cut‐off edge (332, shorter than the one of 400 nm in YVO_4_) for an excellent birefringent material (Figure 5f).^[^ ^59^, ^60^, ^61^ ^]^ Additionally, the powder XRD patterns before and after exposure in air for six months (Figure S12, Supporting Information) indicate that RbHg_5_Br_11_ is air‐stable, which is important for the practical applications.

**Figure 6:**
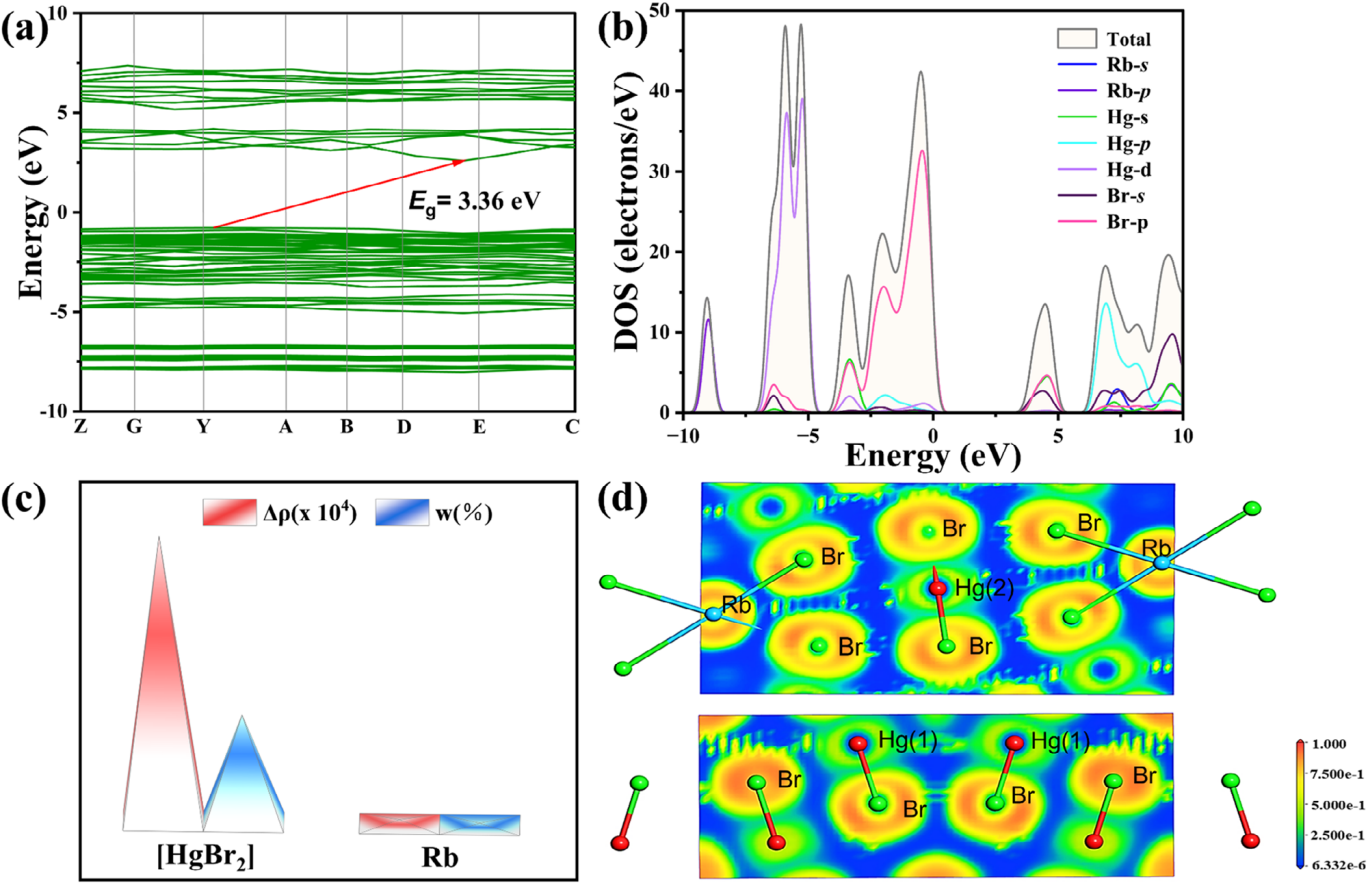
a) The bandgap of RbHg_5_Br_11_; b) Total/partial density of states (T/PDOS); c) The REDA result of RbHg_5_Br_11_; d) Electron‐density difference map of RbHg_5_Br_11_.

To clarify the origin of optical properties, the electronic structures of the six compounds were studied by DFT calculations.^[^ ^62^, ^63^ ^]^ The band structures (**Figure** **6**a; Figure S14, Supporting Information) imply that AHg_5_Br_11_ (A = Rb, Cs), Rb_3_Hg_2_Br_7_ and Rb_3_Hg_2_I_7_ are indirect bandgap compounds with calculated generalized gradient approximation (GGA) bandgaps of 2.55 eV (RbHg_5_Br_11_), 2.63 eV (CsHg_5_Br_11_), 2.86 eV (Rb_3_Hg_2_Br_7_) and 2.67 eV (Rb_3_Hg_2_I_7_), while others are direct bandgap semiconductors with GGA bandgaps of 2.56 eV (Rb_7_Hg_3_Br_13_) and 2.88 eV (RbHg_2_Br_5_). The GGA values are usually lower than the experimental results, which is expected due to the known discontinuity of the exchange‐correlation energy functional.^[^ ^64^ ^]^ To improve the calculation accuracy, we employed the Heyd‐Scuseria‐Ernzerhof (HSE) functional, yielding the HSE06 bandgaps of 3.36 eV for RbHg_5_Br_11_, 3.42 eV for CsHg_5_Br_11_, 3.68 eV for Rb_7_Hg_3_Br_13_, 3.58 eV for Rb_3_Hg_2_Br_7_, 3.29 eV for Rb_3_Hg_2_I_7_, agreeing well with the experimental data. In RbHg_5_Br_11_, the total/partial density of states (T/PDOSs) (Figure 6b) indicates that the valence band maximum (VBM) is mainly occupied by Hg‐5*p*, Hg‐5*d*, and Br‐4*p* orbits; while the conduction band minimum (CBM) is determined by Hg‐5*s* and Br‐4*p* orbits. It implies that the optical bandgap mainly originates from the linear [HgBr_2_] units in AHg_5_Br_11_ (A = Rb, Cs), similar to the cases of planar [Hg_2_Br_5_], tetrahedral [HgBr_4_] units determined bandgaps in the residual compounds (Figure S15, Supporting Information). Moreover, the response electron distribution anisotropy (REDA) analysis (Figure 6c) further confirms that linear [HgBr_2_] units determine its ∆*n* in RbHg_5_Br_11_. Meanwhile, the charge distribution around Hg atoms is highly anisotropic in the electron‐density difference map (Figure 6d). The localized electrons are also beneficial to induce strong optical anisotropy,^[^ ^21^ ^]^ demonstrating that the linear [HgX_2_] is an advantageous structural unit for the design of high performant birefringent materials.

## 3 Conclusion

In summary, this study demonstrates that targeting linear Hg‐halide coordination units enables the discovery of a novel high performance birefringent crystal RbHg_5_Br_11_, synthesized via simple solution growth. The compound exhibits a large ∆*n* of 0.35@546 nm and a wide bandgap of ≈3.7 eV, attributable to the strong polarizability anisotropy of linear [HgBr_2_] units, as well as a good crystal growth habit by solution method. It means that RbHg_5_Br_11_ is a promising UV birefringent crystals for the optical modulation in laser systems. In addition, the results confirm a clear geometric evolution of [HgBr_n_] units as the Rb/Hg ratio increases from 0.2 to 2.33. Specifically, the Hg‐Br coordination geometry transitioned from a linear [HgBr_2_] in RbHg_5_Br_11_ (Rb/Hg = 0.2), through a planar [HgBr_3_] in RbHg_2_Br_5_ (Rb/Hg = 0.5), to a tetrahedral [HgBr_4_] in Rb_7_Hg_3_Br_13_ (Rb/Hg = 2.33), highlighting the elemental ratio regulated fundamental structural motifs in the Hg‐based halides. Both the experimental and theoretical results confirm that the linear [HgX_2_] is an advantageous functional unit for engineering anisotropic functional materials, with RbHg_5_Br_11_ emerging as a promising UV birefringent crystal.

## 4 Experimental Section

### Reagents

All the raw materials, including RbBr (99.99%), RbI (99.99%), CsBr (99.99%), HgBr_2_ (99.99%) and HgI_2_ (99.99%), were purchased from Shanghai Aladdin Biochemistry Technology Co., and used as the starting precursors for the chemical syntheses of RbHg_5_Br_11_, CsHg_5_Br_11_, RbHg_2_Br_5_, Rb_7_Hg_3_Br_13_, Rb_3_Hg_2_Br_7_ and Rb_3_Hg_2_I_7_ without further purifications.

### The Chemical Syntheses and Single Crystal Growth

Crystals of AHg_5_Br_11_ (A = Rb, Cs), RbHg_2_Br_5,_ and Rb_7_Hg_3_Br_13_ were synthesized from an ethanol solution. First, 1.0000 g mixtures with a molar ratio of ABr and HgBr_2_ = 1: 5 (AHg_5_Br_11_), RbBr and HgBr_2_ = 1: 2 (RbHg_2_Br_5_), RbBr and HgBr_2_ = 7: 3 (Rb_7_Hg_3_Br_13_) were weighed, and dissolved in 20 mL ethanol solution. The solvent was naturally evaporated at room temperature.

Rb_3_Hg_2_Br_7_ and Rb_3_Hg_2_I_7_ crystals were grown by the high‐temperature solution method in vacuum‐sealed quartz tubes. First, 1.0000 g mixtures with a molar ratio of RbBr and HgBr_2_ = 3: 2 (Rb_3_Hg_2_Br_7_) and RbI and HgI_2_ = 3: 2 (Rb_3_Hg_2_I_7_) were weighted and mixed in an agate mortar. After that, the samples were sealed in the quartz tubes by methane‐oxygen flame under a vacuum of 10^−3^ Pa. The sealed samples were heated to 450 °C at a rate of 10 °C h^−1^ from the room temperature, and held for 24 h; then heated to 600 °C in 40 h, held for 3 days; cooled to room temperature at a rate of 4 °C h^−1^. The crystals were harvested in the resulting products. For RbHg_5_Br_11_, 2.0000 g of starting materials yielded 1.9177 g crystalline product, corresponding to a yield of 92.36%.

### Structure Determinations

The high quality single crystals were picked under an optical microscope for single‐crystal X‐ray diffraction measurements. Single‐crystal X‐ray diffraction data were collected at room temperature on an APEX II CCD diffractometer using monochromatic Mo K*_α_* radiation and integrated with the SAINT program.^[^ ^65^ ^]^ The crystal structures were determined through the direct methods and refined by full‐matrix least‐squares fitting on *F* ^2^ using SHELXL.^[^ ^66^ ^]^ The program PLATON was used for verifying possible missing symmetry elements, but no higher symmetries were found. The crystal data and refined parameters, atomic coordinates, equivalent isotropic displacement parameters, selected bond lengths and angles of the six compounds are provided in Tables S2–S25 (Supporting Information), respectively.^[^ ^67^ ^]^

### Elemental Analysis

Elemental analyses were characterized on a field‐emission scanning electron microscope (FE‐SEM JEOL JSM‐7610F Plus, Japan) with an energy‐dispersive system (OXFORDD X‐Max 50). The operating voltages were set to 4.0–6.0 kV.

### XRD Measurements

The powder XRD patterns were collected on a Bruker D2 bit‐phase diffractometer (Germany) with Cu K*_α_* radiation (*λ* = 1.5418 Å). The diffraction data were recoded at room temperature with 2*θ* ranges from 5° to 70° and a scanning speed of 0.02° s^−1^. The theoretical XRD patterns of these compounds were generated from the corresponding cif documents by Mercury software.

### UV–Vis‐Near Diffuse‐Reflectance Spectral and Spectroscopic Measurements

Diffuse‐reflectance spectra of the halides were measured on a Shimadzu SolidSpec‐3700DUV spectrophotometer in the wavelength range of 175–2600 nm at room temperature.^[^ ^68^ ^]^ To accurately assess their ultraviolet cut‐off edge, the UV–vis‐IR transmittance spectra were also collected from 175 to 2600 nm using the same equipment.

### Raman Spectra

The Raman spectra of the single crystal were collected in the 4000–100 cm^−1^ (2.5–100 µm) on LABRAM HR Evolution spectrometer equipped with a CCD detector by 532 nm radiation.

### RID Measurements

The RIDs of RbHg_5_Br_11_, CsHg_5_Br_11_ and RbHg_2_Br_5_ were characterized using the Carl Zeiss Axioscope5 polarizing microscope with a Berek compensator. The wavelength used by the light source was 546 nm. The optical path difference (R) in a specific direction was ascertained based on the interference color exhibited by the crystal at its maximum value under polarized light.

### Theoretical Calculations

The electronic structures and optical properties of these six compounds were calculated based on the DFT calculations by the CASTEP package.^[^ ^69^, ^70^, ^71^, ^72^, ^73^ ^]^ The GGA was adopted, and a Perdew‐Burke‐Ernzerhof functional was chosen to calculate the exchange‐correlation potential with an energy cutoff of 880 eV.^[^ ^74^ ^]^ Meanwhile, the Monkhorst‐Pack *k*‐point in the Brillouin zone was set as 0.025 Å^−1^.^[^ ^75^ ^]^ After that, the norm‐conserving pseudopotentials were used to describe the interactions between the ionic core and electron with the following valence electron configurations: Rb 3*d* ^10^4*p* ^6^5*s* ^1^, Cs 4*d* ^10^5*p* ^6^6*s* ^1^, Hg 5*p* ^6^5*d* ^10^6*s* ^2^, Br 3*d* ^10^4*s* ^2^4*p* ^5^, I 4*d* ^10^5*s* ^2^5*p* ^5^. The calculations of the linear optical performance were described in terms of the complex dielectric constant *ε*(*ω*) = *ε*_1_(*ω*) + *iε*_2_(*ω*). And the momentum matrix elements between the occupied and unoccupied electronic states were used to obtain the imaginary part *ε*_2_(*ω*) of the dielectric function *ε*(*ω*):^[^ ^76^, ^77^ ^]^

(1) $$\varepsilon_{2}\left(\hbar \omega \right)=\frac{2e^{2}\pi }{\Omega \varepsilon_{0}}\sum_{kcv}{\left|\langle \psi_{k}^{c}\left|\hat{u}\cdot r\right|\psi_{k}^{v}\rangle \right|}^{2}\delta \left[E_{k}^{c}-E_{k}^{v}-E\right]$$

here, *Ω* is the unit cell volume, *ν* and 𝑐 represent the VB and CB, respectively. ω and 𝑢̂ are the frequency and the unit vector in the polarization direction of the incident light.^[^ ^78^ ^]^ Under the periodic boundary condition, $|\left\langle \psi_{k}^{c}\left|\hat{u}\cdot r\right|\psi_{k}^{v}\right\rangle |$ is the transition matrix element between the VB and the CB at a specific *k* point in the first Brillouin zone. The real part *ε*_1_(*ω*) can be obtained from the imaginary part *ε*_2_(*ω*) using the Kramers–Kronig transformation. Subsequently, the refractive indices (*n*) and ∆*n* can be obtained from the complex dielectric function. Meanwhile, the HOMO‐LUMO gap, polarizability anisotropy, and hyperpolarizability of Hg‐based units were investigated by Gaussian 09 package under the condition of the B3LYP level with LANL2DZ basis sets.

[CCDC 2475643–2475648 contains the supplementary crystallographic data for this paper. These data can be obtained from The Cambridge Crystallographic Data Centre via https://www.ccdc.cam.ac.uk/data%20request/cif.

## Conflict of Interest

The authors declare no conflict of interest.

## Supporting information



Supporting Information

Supporting cif

## Data Availability

The data that support the findings of this study are available in the supplementary material of this article.
