# Supplementary material for: [HgX2] Linear Group Enabled Ultraviolet Birefringent Crystal RbHg5Br11 with Strong Optical Anisotropy and Wide Bandgap
Source: Adv Sci (Weinh). 2025 Sep 9;12(45):e14304. doi: 10.1002/advs.202514304 (PMC12677596; doi:10.1002/advs.202514304)
Supplement: Supplementary file 1 — Supporting Information [file ADVS-12-e14304-s001.docx]

Supporting Information

**[HgX_2_] linear group enabled ultraviolet birefringent crystal RbHg_5_Br_11_ with strong optical anisotropy and wide band gap**

*Yinxia Du, Wangfei Che, Yunfei Shi, Juanjuan Lu, Xiaodong Zhou, Ting Liu*, Shilie Pan*, Junjie Li**

Y. X. Du, W. F. Che, Y. F. Shi, X. D. Zhou, T. Liu

State Key Laboratory of Chemistry and Utilization of Carbon-Based Energy Resources, College of Chemistry, Xinjiang University, Urumqi 830017, China

E-mail: *[liut@xju.edu.cn](mailto:liut@xju.edu.cn)

J. J. Lu, S. L. Pan, J. J. Li

Research Center for Crystal Materials; State Key Laboratory of Functional Materials and Devices for Special Environmental Conditions; Xinjiang Key Laboratory of Functional Crystal Materials; Xinjiang Technical Institute of Physics & Chemistry, Chinese Academy of Sciences, 40-1 South Beijing Road, Urumqi 830011, China

E-mails: *slpan@ms.xjb.ac.cn; [*lijunjie@ms.xjb.ac.cn](mailto:*lijunjie@ms.xjb.ac.cn)

S. L. Pan, J. J. Li

Center of Materials Science and Optoelectronics Engineering University of Chinese Academy of Sciences, Beijing 100049, China

**Table of Contents**

**Table S1.** Structure and optical properties of the typical birefringent materials and Hg-based halide compounds.

**Table S2.** Atomic coordinates (× 10^4^), and equivalent isotropic displacement parameters (*U*_eq_^a^, Å^2^ × 10^3^) for RbHg_5_Br_11_. *U*_eq_ is defined as one third of the trace of the orthogonalized *U*_ij_ tensor.

**Table S3.** Anisotropic displacement parameters (Å^2^ × 10^3^) for RbHg_5_Br_11_. The anisotropic displacement factor exponent takes the form: -2π^2^[h^2^a^*2^U_11_+2hka^*^b^*^U_12_+…].

**Table S4.** Bond lengths for RbHg_5_Br_11_.

**Table S5.** Bond angles for RbHg_5_Br_11_.

**Table S6.** Atomic coordinates (× 10^4^), and equivalent isotropic displacement parameters (*U*_eq_^a^, Å^2^ × 10^3^) for CsHg_5_Br_11_. *U*_eq_ is defined as one third of the trace of the orthogonalized *U*_ij_ tensor.

**Table S7.** Anisotropic displacement parameters (Å^2^ × 10^3^) for CsHg_5_Br_11_. The anisotropic displacement factor exponent takes the form: -2π^2^[h^2^a^*2^U_11_+2hka^*^b^*^U_12_+…].

**Table S8.** Bond lengths for CsHg_5_Br_11_.

**Table S9.** Bond angles for CsHg_5_Br_11_.

**Table S10.** Atomic coordinates (× 10^4^), and equivalent isotropic displacement parameters (*U*_eq_^a^, Å^2^ × 10^3^) for RbHg_2_Br_5_. *U*_eq_ is defined as one third of the trace of the orthogonalized *U*_ij_ tensor.

**Table S11.** Anisotropic displacement parameters (Å^2^ × 10^3^) for RbHg_2_Br_5_. The anisotropic displacement factor exponent takes the form: -2π^2^[h^2^a^*2^U_11_+2hka^*^b^*^U_12_+…].

**Table S12.** Bond lengths for RbHg_2_Br_5_.

**Table S13.** Bond angles for RbHg_2_Br_5_.

**Table S14.** Atomic coordinates (× 10^4^), and equivalent isotropic displacement parameters (*U*_eq_^a^, Å^2^ × 10^3^) for Rb_7_Hg_3_Br_13_. *U*_eq_ is defined as one third of the trace of the orthogonalized *U*_ij_ tensor.

**Table S15.** Anisotropic displacement parameters (Å^2^ × 10^3^) for Rb_7_Hg_3_Br_13_. The anisotropic displacement factor exponent takes the form: -2π^2^[h^2^a^*2^U_11_+2hka^*^b^*^U_12_+…].

**Table S16.** Bond lengths for Rb_7_Hg_3_Br_13_.

**Table S17.** Bond angles for Rb_7_Hg_3_Br_13_.

**Table S18.** Atomic coordinates (× 10^4^), and equivalent isotropic displacement parameters (*U*_eq_^a^, Å^2^ × 10^3^) for Rb_3_Hg_2_Br_7_. *U*_eq_ is defined as one third of the trace of the orthogonalized *U*_ij_ tensor.

**Table S19.** Anisotropic displacement parameters (Å^2^ × 10^3^) for Rb_3_Hg_2_Br_7_. The anisotropic displacement factor exponent takes the form: -2π^2^[h^2^a^*2^U_11_+2hka^*^b^*^U_12_+…].

**Table S20.** Bond lengths for Rb_3_Hg_2_Br_7_.

**Table S21.** Bond angles for Rb_3_Hg_2_Br_7_.

**Table S22.** Atomic coordinates (× 10^4^), and equivalent isotropic displacement parameters (*U*_eq_^a^, Å^2^ × 10^3^) for Rb_3_Hg_2_I_7_. *U*_eq_ is defined as one third of the trace of the orthogonalized *U*_ij_ tensor.

**Table S23.** Anisotropic displacement parameters (Å^2^ × 10^3^) for Rb_3_Hg_2_I_7_. The anisotropic displacement factor exponent takes the form: -2π^2^[h^2^a^*2^U_11_+2hka^*^b^*^U_12_+…].

**Table S24.** Bond lengths for Rb_3_Hg_2_I_7_.

**Table S25.** Bond angles for Rb_3_Hg_2_I_7_.

**Figure of Contents**

**Figure S1.** The results of element analyses for RbHg_5_Br_11_, CsHg_5_Br_11_, RbHg_2_Br_5_, Rb_7_Hg_3_Br_13_, Rb_3_Hg_2_Br_7_ and Rb_3_Hg_2_I_7_.

**Figure S2.** Crystal structure of CsHg_5_Br_11_.

**Figure S3.** The Raman spectrum of RbHg_5_Br_11_.

**Figure S4.** Crystal structure of RbHg_2_Br_5_.

**Figure S5.** Crystal structure of Rb_7_Hg_3_Br_13_.

**Figure S6.** Crystal structure of Rb_3_Hg_2_Br_7_.

**Figure S7.** Crystal structure of Rb_3_Hg_2_I_7_.

**Figure S8.** Ternary diagram for the Rb-Hg-Br system.

**Figure S9.** The calculated birefringence for the series of compounds (CsHg_5_Br_11_, RbHg_2_Br_5_, Rb_7_Hg_3_Br_13_, Rb_3_Hg_2_Br_7_ and Rb_3_Hg_2_I_7_).

**Figure S10.** The experimental band gaps of RbHg_5_Br_11_, Rb_7_Hg_3_Br_13_, Rb_3_Hg_2_Br_7_ and Rb_3_Hg_2_I_7_.

**Figure S11.** a, e) The rotation of compensatory; b, f) Photographs of CsHg_5_Br_11_ and RbHg_2_Br_5_ crystal; c, g) The thicknesses of CsHg_5_Br_11_ and RbHg_2_Br_5_ for the RID measurements; d, h) The crystal orientations of CsHg_5_Br_11_ and RbHg_2_Br_5_ single crystals indexed by single-crystal XRDs.

**Figure S12.** The powder XRD patterns for RbHg_5_Br_11_ before and after exposure in air for six months, the theoretical results utilized as the reference.

**Figure S13.** The powder XRD patterns of Rb_7_Hg_3_Br_13_ (a), Rb_3_Hg_2_Br_7_ (b) and Rb_3_Hg_2_I_7_ (c) polycrystalline pure phase samples.

**Figure S14.** The band structures of CsHg_5_Br_11_ (a), RbHg_2_Br_5_ (b), Rb_7_Hg_3_Br_13_ (c), Rb_3_Hg_2_Br_7_ (d) and Rb_3_Hg_2_I_7_ (e).

**Figure S15.** The total/partial density of states (T/PDOS) of CsHg_5_Br_11_ (a), RbHg_2_Br_5_ (b), Rb_7_Hg_3_Br_13_ (c), Rb_3_Hg_2_Br_7_ (d) and Rb_3_Hg_2_I_7_ (e).

**Table S1.** Structure and optical properties of the typical birefringent materials and Hg-based halide compounds.

| Compound | Space groups | *E*_g_ | Δ*n* | Ref. |
| --- | --- | --- | --- | --- |
| RbHg_5_Br_11_ | *C*2/*m* | 3.73^E^ | 0.259@546 nm^E^ | This work |
|  |  |  | 0.322@1064 nm^C^ |  |
| CsHg_5_Br_11_ | *C*2/*m* | 2.63^C^ | 0.198@546 nm^E^  0.309@1064 nm^C^ | This work |
| RbHg_2_Br_5_ | *P*2_1_/*c* | 3.38^C^ | 0.114@546 nm^E^  0.192@1064 nm^C^ | This work |
| Rb_3_Hg_2_Br_7_ | *P*2_1_/*c* | 3.38^E^ | 0.009@1064 nm^C^ | This work |
| Rb_3_Hg_2_I_7_ | *P*2_1_/*c* | 2.76^E^ | 0.028@1064 nm^C^ | This work |
| Rb_7_Hg_3_Br_13_ | *Pnma* | 3.52^E^ | 0.013@1064 nm^C^ | This work |
| YVO_4_ | *P*4_1_/*amd* | 3.10^E^ | 0.225@532 nm^C^ | [1] |
| LiNbO_3_ | *R*3*c* | 2.95^E^ | 0.084@633 nm^E^ | [2] |
| TiO_2_ | *P*4_2_/*mnm* | 3.10^E^ | 0.256@546 nm^C^ | [3] |
| CaCO_3_ | *R*3*c* | 5.39^E^ | 0.172@532 nm^E^ | [4] |
| *α*-BaB_2_O_4_ | *R*$\bar{3}$*c* | 6.56^E^ | 0.122@546 nm^E^ | [5] |
| MgF_2_ | *P*4_2_/*mnm* | 11.27 ^E^ | 0.012@546 nm^E^ | [6] |
| *α*-SnF_2_ | *C*2/*c* | 4.0^E^ | 0.177@546 nm^E^ | [7] |
| SnCl_2_ | *Pnma* | 3.32^E^ | 0.210@546 nm^C^ | [7] |
| PbCl_2_ | *Pnma* | 4.59^C^ | 0.046@546 nm^E^ | [7] |
| SbCl_3_ | *Pnma* | 4.51^C^ | 0.172@546 nm^E^ | [7] |
| MgCl_2_ | *R*3*m* | 6.99^C^ | 0.024@546 nm^C^ | [7] |
| CaCl_2_ | *Pnma* | 6.96^C^ | 0.028@546 nm^C^ | [7] |
| BaCl_2_ | *Pnma* | 6.36^C^ | 0.010@546 nm^C^ | [7] |
| BiCl_3_ | *Pn*2_1_*a* | 4.43^C^ | 0.056@546 nm^C^ | [7] |
| PbF_2_ | *Pnma* | 5.24^C^ | 0.017@546 nm^C^ | [7] |
| SbF_2_ | *Ama*2 | 5.53^C^ | 0.104@546 nm^C^ | [7] |
| BiF_2_ | *Pnma* | 5.77^C^ | 0.034@546 nm^C^ | [7] |
| Hg_2_Cl_2_ | *I4/mmm* | - | 0.55^C^ | [8] |
| Hg_2_Br_2_ | *I4/mmm* | - | 0.97@1000 nm^C^ | [9] |
| Hg_2_I_2_ | *I4/mmm* | - | 0.96^C^ | [10] |
| HgBr_2_ | *Cmc*2_1_ | 3.6^E^ | 0.235@546 nm^E^ | [11] |
|  |  |  | 0.245@546 nm^C^ |  |
| *β*-CsHg_2_Cl_5_ | *P*2_1_/*m* | 3.54^E^ | 0.29@546 nm^E^ | [12] |
|  |  |  | 0.22@1064 nm^C^ |  |
| CsHgI_2_Cl_2_ | *P*2_1_ | 3.15^E^ | 0.198@1064 nm^C^ | [13] |
| Cs_2_Hg_3_I_8_·H_2_O | *C*_1_*m*_1_ | 2.59^E^ | [0.08@1000](mailto:0.08@1000) nm^E^ | [14] |
| *β*-CsHg_2_I_5_ | *P*2_1_/*c* | 2.58^E^ | [0.132@1064](mailto:0.132@1064) nm^C^ | [15] |
| Cs_2_Hg_2_Br_2_I_4_·H_2_O | *P*_1_*c*_1_ | 2.82^E^ | 0.1@1000 nm^C^ | [16] |
| RbHgI_3_ | *Ama*2 | 2.56^E^ | [0.08@1000](mailto:0.08@1000) nm^C^ | [17] |
| RbHgI_3_·H_2_O | *C*_1_*c*_1_ | 2.60^E^ | [0.05@1064](mailto:0.05@1064) nm^C^ | [18] |
|  | *P*_1_*c*_1_ | 2.45^E^ | 0.055@1064 nm^C^ |  |
| NH_4_HgBr_3_·H_2_O | *Cmm*2 | 3.40^E^ | [0.183@1064](mailto:0.183@1064) nm^C^ | [19] |

^E^: Experimental; ^C^: Calculated.

**Table S2.** Atomic coordinates (× 10^4^), and equivalent isotropic displacement parameters (*U*_eq_^a^, Å^2^ × 10^3^) for RbHg_5_Br_11_. *U*_eq_ is defined as one third of the trace of the orthogonalized *U*_ij_ tensor.

| Atom | x | y | z | *U*_eq_^a^*/*Å^2^ |
| --- | --- | --- | --- | --- |
| Hg1 | 3158.2(2) | -1663.4(2) | 8169.7(4) | 44.71(16) |
| Hg2 | 0 | 0 | 5000 | 41.46(18) |
| Br1 | 5000 | 0 | 10000 | 31.3(2) |
| Br2 | 2035.2(5) | -1242.4(3) | 4257.4(9) | 34.17(18) |
| Br3 | 4227.5(5) | -2191.6(4) | 12018.6(9) | 37.19(19) |
| Br4 | -1440.6(8) | 0 | 1016.0(14) | 43.4(2) |
| Rb1 | 5000 | 0 | 5000 | 63.5(4) |

^a^*U*_eq_ is defined as one third of the trace of the orthogonalized *U*_ij_ tensor.

**Table S3.** Anisotropic displacement parameters (Å^2^ × 10^3^) for RbHg_5_Br_11_. The anisotropic displacement factor exponent takes the form: -2π^2^[h^2^a^*2^U_11_+2hka^*^b^*^U_12_+…].

| Atom | U_11_ | U_22_ | U_33_ | U_23_ | U_13_ | U_12_ |
| --- | --- | --- | --- | --- | --- | --- |
| Hg1 | 37.9(2) | 64.4(2) | 26.7(2) | 13.17(10) | 11.60(14) | 6.02(10) |
| Hg2 | 37.1(3) | 47.4(3) | 26.4(3) | 0 | 4.7(2) | 0 |
| Br1 | 35.0(5) | 30.3(5) | 29.2(5) | 0 | 16.1(4) | 0 |
| Br2 | 41.1(3) | 30.9(3) | 23.9(3) | 2.12(19) | 10.6(2) | 4.8(2) |
| Br3 | 30.8(3) | 50.5(3) | 25.5(3) | 8.6(2) | 9.8(2) | -3.3(2) |
| Br4 | 34.2(4) | 65.7(6) | 24.4(4) | 0 | 9.6(3) | 0 |
| Rb1 | 108.4(13) | 54.8(7) | 49.3(8) | 0 | 55.7(9) | 0 |

**Table S4.** Bond lengths for RbHg_5_Br_11_.

| Atom | Length/ Å |
| --- | --- |
| Hg1 Br3 | 2.4312(6) |
| Hg1 Br2 | 2.4205(6) |
| Hg2 Br4 | 2.4192(9) |
| Hg2 Br4^2^ | 2.4192(9) |
| Br3 Rb1^1^ | 3.7623(7) |
| Br1 Rb1 | 3.4101(3) |
| Br1 Rb1^1^ | 3.4101(3) |
| Br2 Rb1 | 3.9083(6) |

1+X, +Y, 1+Z; 2-X, -Y, 1-Z

**Table S5.** Bond angles for RbHg_5_Br_11_.

| Atom | Angle/° | | Atom | Angle/° |
| --- | --- | --- | --- | --- |
| Br2 Hg1 Br3 | 175.93(2) | Br3^5^ Rb1 Br2^9^ | | 62.041(12) |
| Br2^9^ Rb1 Br2^8^ | 180.0 | Br3^6^ Rb1 Br2 | | 67.752(12) |
| Br3^4^ Rb1 Br2 | 62.040(12) | Br1 Rb1 Br3^3^ | | 65.284(19) |
| Br2^7^ Rb1 Br2^8^ | 57.4883(16) | Br1^4^ Rb1 Br3^6^ | | 114.716(9) |
| Br4^2^ Hg2 Br4 | 180.0 | Br1 Rb1 Br3^6^ | | 65.284(9) |
| Br3^3^ Rb1 Br3^4^ | 180.0 | Br1^4^ Rb1 Br3^4^ | | 65.284(9) |
| Br3^4^ Rb1 Br3^5^ | 123.570(17) | Br1^4^ Rb1 Br3^3^ | | 114.716(9) |
| Br3^3^ Rb1 Br3^5^ | 56.430(17) | Br1^4^ Rb1 Br3^5^ | | 65.284(9) |
| Br3^4^ Rb1 Br3^6^ | 56.430(17) | Br1 Rb1 Br3^5^ | | 114.716(9) |
| Br3^3^ Rb1 Br3^6^ | 123.570(17) | Br1 Rb1 Br3^4^ | | 114.716(9) |
| Br3^5^ Rb1 Br3^6^ | 180.000(17)) | Br1^4^ Rb1 Br1 | | 180.0 |
| Br3^3^ Rb1 Br2^7^ | 62.041(12) | Br1 Rb1 Br2^9^ | | 71.189(9) |
| Br3^6^ Rb1 Br2^7^ | 112.248(12) | Br1^4^ Rb1 Br2^7^ | | 71.189(9) |
| Br3^4^ Rb1 Br2^8^ | 67.752(12) | Br1^4^ Rb1 Br2^8^ | | 71.189(9) |
| Br3^6^ Rb1 Br2^8^ | 62.041(12) | Br1^4^ Rb1 Br2^9^ | | 108.811(9) |
| Br3^3^ Rb1 Br2^9^ | 67.752(12) | Br1 Rb1 Br2^7^ | | 108.811(9) |
| Br3^4^ Rb1 Br2^7^ | 117.959(12) | Br1 Rb1 Br2^8^ | | 108.811(9) |
| Br3^5^ Rb1 Br2^8^ | 117.959(12) | Br1 Rb1 Br2 | | 71.189(9) |
| Br3^4^ Rb1 Br2^9^ | 112.248(12) | Br1^4^ Rb1 Br2 | | 108.811(9) |
| Br3^6^ Rb1 Br2^9^ | 117.959(12) | Br2^7^ Rb1 Br2^9^ | | 122.517(16) |
| Br3^3^ Rb1 Br2^8^ | 112.248(12) | Br2^9^ Rb1 Br2 | | 57.484(16) |
| Br3^5^ Rb1 Br2 | 112.248(12) | Br2^7^ Rb1 Br2 | | 180.0 |
| Br3^3^ Rb1 Br2 | 117.960(12) | Br2^8^ Rb1 Br2 | | 122.516(16) |
| Br3^5^ Rb1 Br2^7^ | 67.752(12) |  | |  |

^1^+X, +Y, 1+Z; ^2^-X, -Y, 1-Z; ^3^1-X, -Y, 2-Z; ^4^+X, +Y, -1+Z; ^5^+X, -Y, -1+Z; ^6^1-X, +Y, 2-Z; ^7^1-X, -Y, 1-Z; ^8^1-X, +Y, 1-Z; ^9^+X, -Y, +Z

**Table S6.** Atomic coordinates (× 10^4^), and equivalent isotropic displacement parameters (*U*_eq_^a^, Å^2^ × 10^3^) for CsHg_5_Br_11_. *U*_eq_ is defined as one third of the trace of the orthogonalized *U*_ij_ tensor.

| Atom | x | y | z | *U*_eq_^a^*/*Å^2^ |
| --- | --- | --- | --- | --- |
| Hg1 | 3155.9(5) | 6664.7(5) | 3178.5(10) | 45.8(3) |
| Hg2 | 0 | 5000 | 0 | 42.7(4) |
| Br1 | 2019.9(12) | 6246.3(10) | -703(2) | 36.4(4) |
| Br2 | 4228.5(12) | 7202.8(11) | 6976(2) | 36.0(4) |
| Br3 | 5000 | 5000 | 5000 | 28.9(6) |
| Br4 | 1446.0(18) | 5000 | 3957(3) | 45.3(6) |
| Cs1 | 5000 | 5000 | 0 | 51.3(6) |

**Table S7.** Anisotropic displacement parameters (Å^2^ × 10^3^) for CsHg_5_Br_11_. The anisotropic displacement factor exponent takes the form: -2π^2^[h^2^a^*2^U_11_+2hka^*^b^*^U_12_+…].

| Atom | U_11_ | U_22_ | U_33_ | U_23_ | U_13_ | U_12_ |
| --- | --- | --- | --- | --- | --- | --- |
| Hg1 | 32.5(4) | 32.5(4) | 30.9(4) | -14.2(3) | 9.7(3) | -7.1(3) |
| Hg2 | 30.3(6) | 48.2(8) | 31.8(6) | 0 | 2.0(5) | 0 |
| Br1 | 33.1(7) | 30.0(8) | 28.7(7) | -1.2(6) | 7.7(6) | -2.8(6) |
| Br2 | 24.8(7) | 45.3(9) | 30.2(7) | -8.5(6) | 7.8(6) | 3.2(6) |
| Br3 | 31.2(13) | 30.6(15) | 26.1(12) | 0 | 15.0(11) | 0 |
| Br4 | 34.2(4) | 65.7(6) | 24.4(4) | 0 | 9.6(3) | 0 |
| Cs1 | 28.1(10) | 68.9(17) | 28.5(10) | 0 | 6.0(8) | 0 |

**Table S8.** Bond lengths for CsHg_5_Br_11_.

| Atom | Length/ Å |
| --- | --- |
| Hg2 Br4^1^ | 2.421(2) |
| Hg2 Br4 | 2.421(2) |
| Hg1 Br2 | 2.4297(16) |
| Hg1 Br1 | 2.4241(16) |
| Br3^2^ Cs1 | 3.4692(8) |
| Br3 Cs1 | 3.4692(8) |
| Br2^3^ Cs1 | 3.8127(18) |
| Br2^4^ Cs1 | 3.8127(18) |
| Br2^2^ Cs1 | 3.8127(18) |
| Br2^5^ Cs1 | 3.8127(18) |
| Br1 Cs1 | 3.9504(16) |
| Br1^6^ Cs1 | 3.9504(16) |
| Br1^7^ Cs1 | 3.9504(16) |
| Br1^8^ Cs1 | 3.9504(16) |

1-X, 1-Y, -Z; 2+X, +Y, -1+Z; 4^1^-X, 1-Y, 1-Z; 6^1^-X, 1-Y, -Z; 7^1^+X, 1-Y, +Z; 8^1^-X, +Y, -Z

**Table S9.** Bond angles for CsHg_5_Br_11_.

| Atom | Angle/° | | Atom | | | Angle/° |
| --- | --- | --- | --- | --- | --- | --- |
| Br4^1^ Hg2 Br4 | 180.0 | Br2^6^ Cs1 Br2^4^ | | | | 57.15(4) |
| Br1^2^ Cs1 Br1^3^ | 180.00(4) | Br2^7^ Cs1 Br2^6^ | | | | 180.0 |
| Br1^3^ Cs1 Br1^8^ | 57.31(5) | Br2^7^ Cs1 Br2^4^ | | | | 122.85(4) |
| Br1 Hg1 Br2 | 175.39(6) | Br2^7^ Cs1 Br1^3^ | | | | 116.89(3) |
| Br2^6^ Cs1 Br2^5^ | 122.85(4) | Br2^4^ Cs1 Br1 | | | | 63.11(3) |
| Br2^7^ Cs1 Br2^5^ | 57.15(4) | Br2^7^ Cs1 Br1 | | | | 112.95(3) |
| Br1 Cs1 Br1^8^ | 180.0 | Br2^5^ Cs1 Br1 | | | | 116.89(3) |
| Br1 Cs1 Br1^3^ | 122.69(5) | Br2^7^ Cs1 Br1^2^ | | | | 63.11(3) |
| Br1^2^ Cs1 Br1^8^ | 122.69(5) | Br2^6^ Cs1 Br1^2^ | | | | 116.89(3) |
| Br3^4^ Cs1 Br3 | 180.0 | Br2^5^ Cs1 Br1^3^ | | | | 112.95(3) |
| Br3 Cs1 Br2^5^ | 64.77(2) | Br2^6^ Cs1 Br1^3^ | | | | 63.11(3) |
| Br3 Cs1 Br2^6^ | 64.77(2) | Br2^5^ Cs1 Br1^8^ | | | | 63.11(3) |
| Br3^4^ Cs1 Br2^4^ | 64.77(2) | Br2^4^ Cs1 Br1^3^ | | | | 67.05(3) |
| Br3^4^ Cs1 Br2^6^ | 115.23(2) | Br2^5^ Cs1 Br1^2^ | | | | 67.05(3) |
| Br3^4^ Cs1 Br2^5^ | 115.23(2) | Br2^7^ Cs1 Br1^8^ | | | | 67.05(3) |
| Br3 Cs1 Br2^4^ | 115.23(2) | Br2^6^ Cs1 Br1 | | | | 67.05(3) |
| Br3 Cs1 Br2^7^ | 115.23(2) | Br2^4^ Cs1 Br1^2^ | | | | 112.95(3) |
| Br3^4^ Cs1 Br2^7^ | 64.77(2) | Br2^6^ Cs1 Br1^8^ | | | | 112.95(3) |
| Br3 Cs1 Br1^3^ | 109.82(2) | Br2^4^ Cs1 Br1^8^ | | | | 116.89(3) |
| Br3 Cs1 Br1^8^ | 109.82(2) | Br1 Cs1 Br1^2^ | | | | 57.31(5) |
| Br3 Cs1 Br1^2^ | 70.18(2) | Br3^4^ Cs1 Br1 | | | | 109.82(2) |
| Br3^4^ Cs1 Br1^3^ | 70.18(2) | Br3^4^ Cs1 Br1^2^ | | | | 109.82(2) |
| Br3 Cs1 Br1 | 70.18(2) | Br3^4^ Cs1 Br1^8^ | | | | 70.18(2) |
| Br2^5^ Cs1 Br2^4^ | 180.00(2) |  | |  |  |  |

^1^-X, 1-Y, -Z; ^2^+X, 1-Y, +Z; ^3^1-X, +Y, -Z; ^4^+X, +Y, -1+Z; ^5^1-X, 1-Y, 1-Z; ^6^1-X, +Y, 1-Z; ^7^+X, 1-Y, -1+Z; ^8^1-X, 1-Y, -Z; ^9^+X, +Y, 1+Z

**Table S10.** Atomic coordinates (× 10^4^), and equivalent isotropic displacement parameters (*U*_eq_^a^, Å^2^ × 10^3^) for RbHg_2_Br_5_. *U*_eq_ is defined as one third of the trace of the orthogonalized *U*_ij_ tensor.

| Atom | x | y | z | *U*_eq_^a^*/*Å^2^ |
| --- | --- | --- | --- | --- |
| Hg1 | 4490.8(7) | 5341.3(6) | 6564.4(5) | 72.5(3) |
| Hg2 | 3341.0(6) | 1415.3(6) | 4638.4(5) | 64.9(2) |
| Br1 | 7170.2(14) | 5434.5(13) | 6227.0(10) | 51.0(3) |
| Br2 | 1619.6(17) | 5556.9(14) | 6525.0(15) | 73.0(5) |
| Br3 | 4132(3) | 23888.6(15) | 6571.8(11) | 86.6(6) |
| Br4 | 6048.3(15) | 1274.2(13) | 4209.5(11) | 55.1(4) |
| Br5 | 317.7(15) | 1087.1(12) | 3838.5(10) | 49.3(3) |
| Rb1 | -382(2) | 2614.2(14) | 1321.4(12) | 73.1(4) |

^a^*U*_eq_ is defined as one third of the trace of the orthogonalized *U*_ij_ tensor.

**Table S11.** Anisotropic displacement parameters (Å^2^ × 10^3^) for RbHg_2_Br_5_. The anisotropic displacement factor exponent takes the form: -2π^2^[h^2^a^*2^U_11_+2hka^*^b^*^U_12_+…].

| Atom | U_11_ | U_22_ | U_33_ | U_23_ | U_13_ | U_12_ |
| --- | --- | --- | --- | --- | --- | --- |
| Hg1 | 39.8(3) | 95.7(5) | 91.1(5) | 7.9(4) | 33.8(3) | 4.1(3) |
| Hg2 | 37.2(3) | 73.0(4) | 78.8(4) | 7.5(3) | 9.2(3) | 0.3(2) |
| Br1 | 30.1(6) | 64.0(8) | 61.2(8) | 6.6(6) | 17.3(5) | 4.0(5) |
| Br2 | 45.8(8) | 60.4(9) | 128.8(14) | -11.1(9) | 50.3(9) | -4.0(6) |
| Br3 | 145.0(17) | 53.1(9) | 44.7(8) | 3.9(6) | 4.4(9) | 0.3(10) |
| Br4 | 41.2(7) | 55.4(8) | 71.7(9) | 29.6(7) | 21.8(6) | 8.7(6) |
| Br5 | 37.8(6) | 54.4(8) | 52.0(7) | 6.7(6) | 8.5(5) | 1.4(5) |
| Rb1 | 89.3(11) | 56.9(9) | 71.0(9) | 17.9(7) | 21.8(8) | 9.9(8) |

**Table S12.** Bond lengths for RbHg_2_Br_5_.

| Atom | Length/ Å | Atom | Length/ Å |
| --- | --- | --- | --- |
| Hg2 Br5 | 2.4595(13) | Br5 Rb1^1^ | 3.7466(19) |
| Hg2 Br4 | 2.5164(13) | Br5 Rb1^2^ | 3.614(2) |
| Hg2 Br3 | 2.6258(16) | Br1 Rb1^4^ | 4.055(2) |
| Hg1 Br1 | 2.4322(12) | Br4 Rb1^3^ | 3.596(2) |
| Hg1 Br2 | 2.4126(13) | Br2 Rb1^1^ | 3.671(2) |
| Hg1 Br3 | 3.0815(17) | Br2 Rb1^5^ | 3.815(2) |
| Br5 Rb1 | 3.5544(19) | Rb1 Br3^6^ | 3.713(3) |

^1^+X, 1/2-Y, 1/2+Z; ^2^-X, -1/2+Y, 1/2-Z; ^3^1+X, 1/2-Y, 1/2+Z; ^4^1-X, 1-Y, 1-Z; ^5^-X, 1-Y, 1-Z; ^6^+X, 1/2-Y, -1/2+Z

**Table S13.** Bond angles for RbHg_2_Br_5_.

| Atom | Angle/° | Atom | Angle/° |
| --- | --- | --- | --- |
| Br5 Hg2 Br4 | 141.77(5) | Br5 Rb1 Br2^6^ | 65.90(4) |
| Br4^8^ Rb1 Br2^6^ | 126.84(6) | Br5^6^ Rb1 Br2^5^ | 128.44(5) |
| Br5 Hg2 Br3 | 112.79(6) | Br5^7^ Rb1 Br2^5^ | 63.83(4) |
| Br4^8^ Rb1 Br1^8^ | 85.75(5) | Br5 Rb1 Br2^5^ | 62.23(4) |
| Br4 Hg2 Br3 | 105.29(6) | Br5^7^ Rb1 Br2^6^ | 152.20(6) |
| Br4^8^ Rb1 Br1^4^ | 130.36(5) | Br5 Rb1 Br3^6^ | 92.67(5) |
| Br1 Hg1 Br3 | 97.89(6) | Br5^7^ Rb1 Br3^6^ | 88.43(5) |
| Br2 Hg1 Br1 | 166.50(6) | Br4^8^ Rb1 Br2^5^ | 93.42(5) |
| Br2 Hg1 Br3 | 89.64(6) | Br4^8^ Rb1 Br3^6^ | 131.09(5) |
| Br2^6^ Rb1 Br5^6^ | 101.19(5) | Br1^8^ Rb1 Br1^4^ | 132.17(4) |
| Br2^6^ Rb1 Br1^4^ | 102.23(5) | Br1^8^ Rb1 Br2^5^ | 99.85(5) |
| Br2^6^ Rb1 Br1^8^ | 58.78(4) | Br5^7^ Rb1 Br5^6^ | 65.22(4) |
| Br2^5^ Rb1 Br1^4^ | 55.11(3) | Br4^8^ Rb1 Br5^6^ | 61.92(4) |
| Br2^6^ Rb1 Br2^5^ | 128.09(4) | Br4^8^ Rb1 Br5^7^ | 70.04(4) |
| Br5 Rb1 Br5^7^ | 119.89(5) | Br2^6^ Rb1 Br3^6^ | 63.79(4) |
| Br5 Rb1 Br5^6^ | 161.49(6) | Br5^7^ Rb1 Br1^4^ | 61.93(4) |
| Br5 Rb1 Br1^8^ | 65.57(4) | Br3^6^ Rb1 Br5^6^ | 69.21(4) |
| Br5^6^ Rb1 Br1^4^ | 105.57(5) | Br3^6^ Rb1 Br1^8^ | 122.56(5) |
| Br5 Rb1 Br1^4^ | 66.63(4) | Br3^6^ Rb1 Br1^4^ | 61.11(4) |
| Br5^6^ Rb1 Br1^8^ | 120.44(5) | Br3^6^ Rb1 Br2^5^ | 116.21(5) |
| Br5 Rb1 Br4^8^ | 136.23(6) | Br5^7^ Rb1 Br1^8^ | 148.93(6) |

^1^+X, 1/2-Y, 1/2+Z; ^2^-X, -1/2+Y, 1/2-Z; ^3^1+X, 1/2-Y, 1/2+Z; ^4^1-X, 1-Y, 1-Z; ^5^-X, 1-Y, 1-Z; ^6^+X, 1/2-Y, -1/2+Z; ^7^-X, 1/2+Y, 1/2-Z; ^8^-1+X, 1/2-Y, -1/2+Z

**Table S14.** Atomic coordinates (× 10^4^), and equivalent isotropic displacement parameters (*U*_eq_^a^, Å^2^ × 10^3^) for Rb_7_Hg_3_Br_13_. *U*_eq_ is defined as one third of the trace of the orthogonalized *U*_ij_ tensor.

| Atom | x | y | z | *U*_eq_^a^*/*Å^2^ |
| --- | --- | --- | --- | --- |
| Hg1 | 4540.2(2) | 4524.5(2) | 6976.6(2) | 45.72(7) |
| Hg2 | 2360.1(2) | 2500 | 2887.3(3) | 42.59(8) |
| Br1 | 421.2(6) | 2500 | 3533.2(9) | 50.0(2) |
| Br2 | 2669.6(5) | 1645.0(2) | 1807.2(8) | 62.36(18) |
| Br3 | 3356.8(6) | 2500 | 5302.3(8) | 45.15(18) |
| Br4 | 6073.5(6) | 2500 | 6729.6(10) | 54.4(2) |
| Br5 | 4129.1(5) | 3774.6(2) | 8721.2(8) | 67.1(2) |
| Br6 | 3390.2(4) | 4314.4(2) | 4850.8(6) | 64.87(14) |
| Br7 | 4239.6(4) | 5390.5(2) | 8135.5(7) | 49.71(14) |
| Br8 | 6390.1(4) | 4309.4(2) | 6425.6(6) | 45.15(13) |
| Rb1 | 5167.8(5) | 3398.3(2) | 4276.6(7) | 59.42(17) |
| Rb2 | 3605.5(6) | 2500 | 8913.0(8) | 51.9(2) |
| Rb3 | 6588.8(5) | 3464.6(2) | 9129.7(8) | 64.59(18) |
| Rb4 | 8125.1(4) | 4666.8(2) | 3837.0(6) | 44.86(13) |

^a^*U*_eq_ is defined as one third of the trace of the orthogonalized *U*_ij_ tensor.

**Table S15.** Anisotropic displacement parameters (Å^2^ × 10^3^) for Rb_7_Hg_3_Br_13_. The anisotropic displacement factor exponent takes the form: -2π^2^[h^2^a^*2^U_11_+2hka^*^b^*^U_12_+…].

| Atom | U_11_ | U_22_ | U_33_ | U_23_ | U_13_ | U_12_ |
| --- | --- | --- | --- | --- | --- | --- |
| Hg1 | 43.52(12) | 40.04(12) | 53.60(14) | -5.59(10) | -2.11(10) | 4.20(9) |
| Hg2 | 48.43(17) | 33.64(15) | \| 45.72(17) \|  \| \| --- \| --- \| | \| 0 \| \| --- \| | \| 0.44(14) \| \| --- \| | 0 |
| Br1 | 38.0(4) | 63.4(5) | 48.6(5) | 0 | 10.3(4) | 0 |
| Br2 | 74.2(4) | 37.2(3) | 75.7(4) | -16.4(3) | -3.9(4) | 12.8(3) |
| Br3 | 40.1(4) | 58.3(5) | 37.0(4) | 0 | -2.5(3) | 0 |
| Br4 | 48.6(5) | 47.7(5) | 67.0(5) | 0 | -15.8(4) | 0 |
| Br5 | 60.9(4) | 46.4(3) | 94.1(5) | 23.7(3) | 25.8(4) | 6.7(3) |
| Br6 | 46.1(3) | 47.8(3) | 46.7(3) | -1.3(2) | -7.4(3) | 3.0(3) |
| Br7 | 46.5(3) | 41.5(3) | 61.1(4) | -11.2(3) | 2.6(3) | 5.8(2) |
| Br8 | 37.1(3) | 45.2(3) | 53.2(3) | 3.6(2) | 10.6(2) | -0.5(2) |
| Rb1 | 53.8(3) | 44.9(3) | 79.5(4) | -13.0(3) | 6.3(3) | 5.1(3) |
| Rb2 | 55.9(5) | 54.6(5) | 45.4(4) | 0 | -10.9(4) | 0 |
| Rb3 | 65.4(4) | 42.5(3) | 85.9(5) | 13.2(3) | -26.4(4) | -1.3(3) |
| Rb4 | 43.3(3) | 43.1(3) | 48.1(3) | 1.3(2) | 4.9(2) | 0.0(2) |

**Table S16.** Bond lengths for Rb_7_Hg_3_Br_13_.

| Atom | Length/ Å | Atom | Length/ Å |
| --- | --- | --- | --- |
| Hg2 Br3 | 2.6500(8) | Br8 Rb1 | 3.5580(9) |
| Hg2 Br1 | 2.6842(9) | Br8 Rb3 | 3.4075(9) |
| Hg2 Br2^6^ | 2.5174(6) | Br6 Rb1 | 3.4519(9) |
| Hg2 Br2 | 2.5174(6) | Br6 Rb3^4^ | 3.4474(9) |
| Hg1 Br8 | 2.6105(6) | Br1 Rb2^4^ | 3.4388(11) |
| Hg1 Br6 | 2.5990(6) | Br1 Rb1^2^ | 3.5819(9) |
| Hg1 Br7 | 2.5725(6) | Br1 Rb1^3^ | 3.5819(9) |
| Hg1 Br5 | 2.6396(7) | Br1 Rb3^5^ | 3.7255(9) |
| Rb4 Br8 | 3.5156(8) | Br1 Rb3^4^ | 3.7255(9) |
| Rb4 Br8^9^ | 3.6014(8) | Rb2 Br4^4^ | 3.4670(13) |
| Rb4 Br6^7^ | 3.6031(8) | Rb2 Br4 | 3.9159(13) |
| Rb4 Br6^10^ | 3.6281(9) | Rb1 Br2^12^ | 3.5268(10) |
| Rb4 Br7^9^ | 3.6172(9) | Rb1 Br2^6^ | 4.1000(11) |
| Rb4 Br7^11^ | 3.7582(9) | Rb3 Br2^15^ | 4.1200(11) |
| Rb4 Br7^7^ | 3.6967(9) | Rb2 Br2^13^ | 3.7689(10) |
| Rb4 Br2^12^ | 3.5775(9) | Rb2 Br2^14^ | 3.7689(10) |
| Rb4 Br5^11^ | 3.5693(10) | Rb2 Br5 | 3.4508(8) |
| Br3 Rb2 | 3.4315(12) | Rb2 Br5^6^ | 3.4508(8) |
| Br3 Rb1 | 3.5429(9) | Br7 Rb1^7^ | 4.0146(10) |
| Br3 Rb1^6^ | 3.5430(9) | Br4 Rb1 | 3.5389(10) |
| Br3 Rb3^5^ | 3.5334(9) | Br4 Rb1^6^ | 3.5390(10) |
| Br4 Rb3^6^ | 3.4859(10) | Br4 Rb3 | 3.4859(10) |
| Br3 Rb3^4^ | 3.5334(9) | Rb3 Br5 | 3.4377(10) |

^1^+X, +Y, -1+Z; ^2^-1/2+X, 1/2-Y, 1/2-Z; ^3^-1/2+X, +Y, 1/2-Z; ^4^-1/2+X, +Y, 3/2-Z; ^5^-1/2+X, 1/2-Y, 3/2-Z; ^6^+X, 1/2-Y, +Z; ^7^1-X, 1-Y, 1-Z; ^8^3/2-X, 1-Y, 1/2+Z; ^9^3/2-X, 1-Y, -1/2+Z; ^10^1/2+X, +Y, 1/2-Z; ^11^1/2+X, +Y, 3/2-Z; ^12^1/2+X, 1/2-Y, 1/2-Z; ^13^+X, 1/2-Y, 1+Z; ^14^+X, +Y, 1+Z; ^15^1/2+X, 1/2-Y, 3/2-Z

**Table S17.** Bond angles for Rb_7_Hg_3_Br_13_.

| Atom | Angle/° | Atom | Angle/° |
| --- | --- | --- | --- |
| Br3 Hg2 Br1 | 107.31(3) | Br2^13^ Rb2 Br4 | 131.871(19) |
| Br2 Hg2 Br3 | 105.410(19) | Br2^14^ Rb2 Br4 | 131.87(2) |
| Br2^6^ Hg2 Br3 | 105.410(19) | Br2^14^ Rb2 Br2^13^ | 73.79(2) |
| Br2^6^ Hg2 Br1 | 104.70(2) | Br5 Rb2 Br4^5^ | 101.078(18) |
| Br2 Hg2 Br1 | 104.70(2) | Br5 Rb2 Br4 | 78.375(18) |
| Br2^6^ Hg2 Br2 | 128.01(4) | Br5^6^ Rb2 Br4^5^ | 101.079(18) |
| Br8 Hg1 Br5 | 99.29(2) | Br5^6^ Rb2 Br4 | 78.374(18) |
| Br6 Hg1 Br8 | 111.64(2) | Br5^6^ Rb2 Br2^14^ | 133.90(3) |
| Br6 Hg1 Br5 | 101.40(2) | Br5 Rb2 Br2^14^ | 61.305(17) |
| Br7 Hg1 Br8 | 115.45(2) | Br5^6^ Rb2 Br2^13^ | 61.305(17) |
| Br7 Hg1 Br6 | 115.23(2) | Br5 Rb2 Br2^13^ | 133.90(3) |
| Br7 Hg1 Br5 | 111.73(2) | Br56 Rb2 Br5 | 155.62(4) |
| Br3 Rb1 Br8 | 128.12(3) | Br3 Rb1 Br2^6^ | 64.609(19) |
| Br3 Rb1 Br1^11^ | 79.84(2) | Br8 Rb1 Br1^11^ | 146.04(2) |
| Br3 Rb1 Br7^7^ | 145.94(2) | Br8 Rb1 Br7^7^ | 72.026(18) |
| Br8 Rb4 Br8^10^ | 139.834(17) | Br8 Rb1 Br2^6^ | 136.44(2) |
| Br8^10^ Rb4 Br6^7^ | 76.002(18) | Br6 Rb1 Br4 | 127.46(3) |
| Br8^10^ Rb4 Br6^11^ | 64.319(17) | Br6 Rb1 Br3 | 87.12(2) |
| Br8 Rb4 Br6^7^ | 65.431(17) | Br6 Rb1 Br8 | 75.857(19) |
| Br8 Rb4 Br6^11^ | 131.75(2) | Br6 Rb1 Br1^11^ | 130.47(3) |
| Br8^10^ Rb4 Br7^10^ | 74.759(16) | Br6 Rb1 Br7^7^ | 70.535(18) |
| Br8^10^ Rb4 Br7^7^ | 82.399(18) | Br6 Rb1 Br2^12^ | 137.09(2) |
| Br8 Rb4 Br7^10^ | 140.34(2) | Br6 Rb1 Br2^6^ | 62.547(18) |
| Br8^10^ Rb4 Br7^9^ | 91.525(19) | Br1^11^ Rb1 Br7^7^ | 95.12(2) |
| Br8 Rb4 Br7^9^ | 82.672(19) | Br6^7^ Rb4 Br6^11^ | 125.40(2) |
| Br8 Rb4 Br7^7^ | 76.510(19) | Br6^7^ Rb4 Br7^10^ | 130.64(2) |
| Br8 Rb4 Br2^12^ | 75.130(18) | Br6^7^ Rb4 Br7^7^ | 73.478(17) |
| Br8 Rb4 Br5^9^ | 67.90(2) | Br6^11^ Rb4 Br7^7^ | 65.746(17) |
| Br1^11^ Rb1 Br2^6^ | 68.73(2) | Br6^11^ Rb4 Br7^9^ | 145.50(2) |
| Br7^7^ Rb1 Br2^6^ | 82.069(18) | Br6^7^ Rb4 Br7^9^ | 65.348(16) |
| Br7^10^ Rb4 Br6^11^ | 73.511(16) | Br4 Rb1 Br3 | 66.89(2) |
| Br7^10^ Rb4 Br7^7^ | 138.821(18) | Br4 Rb1 Br8 | 85.46(2) |
| Br7^10^ Rb4 Br7^9^ | 76.613(19) | Br4 Rb1 Br1^11^ | 90.44(2) |
| Br7^7^ Rb4 Br7^9^ | 138.602(17) | Br4 Rb1 Br7^7^ | 147.16(2) |
| Br2^12^ Rb4 Br8^10^ | 130.79(2) | Br2^12^ Rb1 Br3 | 135.79(2) |
| Br2^12^ Rb4 Br6^7^ | 133.43(2) | Br2^12^ Rb1 Br8 | 75.237(19) |
| Br2^12^ Rb4 Br6^11^ | 66.658(17) | Br2^12^ Rb1 Br1^11^ | 70.829(19) |
| Br2^12^ Rb4 Br7^7^ | 74.113(18) | Br2^12^ Rb1 Br7^7^ | 70.758(18) |
| Br2^12^ Rb4 Br7^9^ | 133.86(2) | Br2^12^ Rb1 Br4 | 80.73(2) |
| Br2^12^ Rb4 Br7^10^ | 95.549(19) | Br2^12^ Rb1 Br2^6^ | 128.24(2) |
| Br5^9^ Rb4 Br8^10^ | 146.99(2) | Br5^9^ Rb4 Br7^7^ | 128.79(2) |
| Br5^9^ Rb4 Br6^11^ | 114.50(2) | Br5^9^ Rb4 Br2^12^ | 62.188(19) |
| Br5^9^ Rb4 Br6^7^ | 119.15(2) | Hg2^9^ Rb3 Br2^5^ | 36.296(11) |
| Br5^9^ Rb4 Br7^10^ | 73.653(19) | Br3^9^ Rb3 Br1^9^ | 72.53(2) |
| Br5^9^ Rb4 Br7^9^ | 72.093(18) | Br3^9^ Rb3 Br2^15^ | 64.456(18) |
| Br4^5^ Rb2 Br2^13^ | 78.33(2) | Br6^9^ Rb3 Br3^9^ | 87.34(2) |
| Br8 Rb3 Br4 | 88.62(2) | Br6^9^ Rb3 Br1^9^ | 125.35(3) |
| Br8 Rb3 Br2^15^ | 140.72(2) | Br8 Rb3 Br3^9^ | 129.88(3) |
| Br8 Rb3 Br5 | 71.532(18) | Br8 Rb3 Br6^9^ | 80.64(2) |
| Br1^9^ Rb3 Br2^15^ | 63.113(19) | Br8 Rb3 Br1^9^ | 149.50(2) |
| Br4 Rb3 Br3^9^ | 72.81(2) | Br6^9^ Rb3 Br4 | 143.15(3) |
| Br4 Rb3 Br1^9^ | 78.49(2) | Br5 Rb3 Br3^9^ | 147.15(2) |
| Br3 Rb2 Br1^9^ | 140.23(3) | Br6^9^ Rb3 Br2^15^ | 62.349(17) |
| Br3 Rb2 Br4 | 63.78(2) | Br4 Rb3 Br2^15^ | 128.78(2) |
| Br3 Rb2 Br4^5^ | 74.30(3) | Br5 Rb3 Br4 | 84.80(2) |
| Br3 Rb2 Br2^13^ | 133.64(2) | Br5 Rb3 Br6^9^ | 123.73(2) |
| Br3 Rb2 Br2^14^ | 133.64(2) | Br5 Rb3 Br1^9^ | 79.78(2) |
| Br3 Rb2 Br5 | 88.15(2) | Br5 Rb3 Br2^15^ | 117.56(3) |
| Br3 Rb2 Br5^6^ | 88.15(2) | Br1^9^ Rb2 Br4 | 76.45(3) |
| Br1^9^ Rb2 Br2^14^ | 74.22(2) | Br1^9^ Rb2 Br4^5^ | 145.48(3) |
| Br1^9^ Rb2 Br2^13^ | 74.22(2) | Br1^9^ Rb2 Br5 | 83.762(19) |
| Br1^9^ Rb2 Br5^6^ | 83.761(19) | Br4^5^ Rb2 Br4 | 138.08(2) |
| Br4^5^ Rb2 Br2^14^ | 78.33(2) |  |  |

^1^+X, +Y, -1+Z; ^2^-1/2+X, 1/2-Y, 1/2-Z; ^3^-1/2+X, +Y, 1/2-Z; ^4^-1/2+X, 1/2-Y, 3/2-Z; ^5^-1/2+X, +Y, 3/2-Z; ^6^+X, 1/2-Y, +Z; ^7^1-X, 1-Y, 1-Z; ^8^3/2-X, 1-Y, 1/2+Z; ^9^1/2+X, +Y, 3/2-Z; ^10^3/2-X, 1-Y, -1/2+Z; ^11^1/2+X, +Y, 1/2-Z; ^12^1/2+X, 1/2-Y, 1/2-Z; ^13^+X, +Y, 1+Z; ^14^+X, 1/2-Y, 1+Z; ^15^1/2+X, 1/2-Y, 3/2-Z

**Table S18.** Atomic coordinates (× 10^4^), and equivalent isotropic displacement parameters (*U*_eq_^a^, Å^2^ × 10^3^) for Rb_3_Hg_2_Br_7_. *U*_eq_ is defined as one third of the trace of the orthogonalized *U*_ij_ tensor.

| Atom | x | y | z | *U*_eq_^a^*/*Å^2^ |
| --- | --- | --- | --- | --- |
| Hg1 | 7027.3(3) | 6813.8(6) | 4971.8(2) | 44.18(13) |
| Hg2 | 7721.6(4) | 7826.2(6) | 2955.4(3) | 54.31(15) |
| Br1 | 7285.2(9) | 8231.1(16) | 6201.2(5) | 45.8(3) |
| Br2 | 4990.2(10) | 7170(2) | 4382.5(7) | 66.1(4) |
| Br3 | 8223.1(10) | 3890.7(14) | 5177.7(5) | 45.9(2) |
| Br4 | 7532.3(9) | 4429.5(13) | 3153.6(5) | 42.7(2) |
| Br5 | 8298.9(11) | 9039.1(16) | 4373.6(5) | 51.6(3) |
| Br6 | 5888.9(9) | 9569.9(16) | 2544.8(6) | 48.7(3) |
| Br7 | 9556.8(9) | 9235.9(14) | 2705.4(5) | 42.9(2) |
| Rb1 | 5657.4(9) | 12082.8(16) | 3956.8(6) | 49.5(3) |
| Rb2 | 9962.4(9) | 2564.7(16) | 3997.3(5) | 47.4(2) |
| Rb3 | 7616.0(10) | 2189.1(16) | 1705.0(5) | 50.4(3) |

^a^*U*_eq_ is defined as one third of the trace of the orthogonalized *U*_ij_ tensor.

**Table S19.** Anisotropic displacement parameters (Å^2^ × 10^3^) for Rb_3_Hg_2_Br_7_. The anisotropic displacement factor exponent takes the form: -2π^2^[h^2^a^*2^U_11_+2hka^*^b^*^U_12_+…].

| Atom | U_11_ | U_22_ | U_33_ | U_23_ | U_13_ | U_12_ |
| --- | --- | --- | --- | --- | --- | --- |
| Hg1 | 37.0(2) | 54.2(3) | 38.4(2) | -0.05(16) | 0.13(16) | 2.40(16) |
| Hg2 | 46.0(3) | 39.3(2) | 77.6(3) | 10.4(2) | 11.8(2) | -2.30(17) |
| Br1 | 39.2(5) | 65.5(7) | 33.0(5) | -11.0(4) | 7.7(4) | -0.6(5) |
| Br2 | 34.7(6) | 101.9(11) | 54.5(7) | 8.4(6) | -8.5(5) | -4.2(6) |
| Br3 | 57.0(6) | 40.3(5) | 43.5(5) | -0.2(4) | 17.6(5) | 7.5(4) |
| Br4 | 48.5(6) | 34.9(5)) | 46.2(5) | 2.8(4) | 12.8(4) | -3.3(4) |
| Br5 | 67.0(7) | 49.6(6) | 39.4(5) | -1.0(4) | 13.1(5) | -23.6(5) |
| Br6 | 41.3(6) | 51.7(6) | 50.5(6) | -6.2(5) | 2.8(4) | 7.0(4) |
| Rb1 | 40.6(6) | 58.5(6) | 53.7(6) | -6.0(5) | 19.9(5) | -5.4(4) |
| Rb2 | 31.4(5) | 58.4(6) | 51.7(6) | -6.7(5) | 6.3(4) | -7.1(4) |
| Rb3 | 56.2(7) | 57.7(6) | 34.0(5) | 1.3(4) | 0.8(4) | 2.6(5) |

**Table S20.** Bond lengths for Rb_3_Hg_2_Br_7_.

| Atom | Length/ Å | Atom | Length/ Å |
| --- | --- | --- | --- |
| Rb2 Br5^1^ | 3.4472(15) | Rb1 Br5 | 3.8223(18) |
| Rb2 Br5^3^ | 3.7391(16) | Rb1 Br2^4^ | 3.945(2) |
| Hg1 Br1 | 2.6632(10) | Rb1 Br2 | 3.824(2) |
| Br3 Rb3^6^ | 3.4118(15) | Rb1 Br2^2^ | 3.6440(18) |
| Hg1 Br3 | 2.5664(11) | Br1 Rb2^3^ | 3.4519(15) |
| Hg1 Br5 | 2.6731(11) | Br1 Rb3^6^ | 4.1023(17) |
| Hg1 Br2 | 2.5138(13) | Br1 Rb3^7^ | 3.5089(17) |
| Hg2 Br4 | 2.5393(11) | Br7 Rb2^8^ | 3.8138(15) |
| Hg2 Br7 | 2.5701(11) | Br7 Rb2^4^ | 3.5440(15) |
| Rb3 Br6^1^ | 3.5015(17) | Br7 Rb3^8^ | 3.6857(16) |
| Hg2 Br6 | 2.5375(12) | Br7 Rb3^4^ | 3.5171(16) |
| Hg2 Br5 | 2.9604(12) | Rb2 Br3 | 3.6036(15) |
| Br4 Rb1^1^ | 3.4790(14) | Rb2 Br3^3^ | 3.5858(16) |
| Br4 Rb2 | 3.3666(16) | Rb1 Br3^4^ | 3.7907(17) |
| Br4 Rb3 | 3.3850(15) | Rb1 Br6^5^ | 3.7185(17) |
| Rb1 Br1^2^ | 3.4874(15) | Rb3 Br2^10^ | 3.4541(16) |
| Rb1 Br6 | 3.4621(15) |  |  |

^1^+X, -1+Y, +Z; ^2^1-X, 2-Y, 1-Z; ^3^2-X, 1-Y, 1-Z; ^4^+X, 1+Y, +Z; ^5^1-X, 1/2+Y, 1/2-Z; ^6^+X, 1/2-Y, 1/2+Z; ^7^+X, 3/2-Y, 1/2+Z; ^8^2-X, 1/2+Y, 1/2-Z; ^9^2-X, -1/2+Y, 1/2-Z; ^10^1-X, -1/2+Y, 1/2-Z

**Table S21.** Bond angles for Rb_3_Hg_2_Br_7_.

| Atom | Angle/° | Atom | Angle/° |
| --- | --- | --- | --- |
| Br1 Hg1 Br5 | 102.41(4) | Br1^3^ Rb2 Br5^3^ | 70.54(3) |
| Br7^1^ Rb2 Br7^9^ | 64.82(2) | Br3 Hg1 Br1 | 102.39(4) |
| Br7^1^ Rb2 Br3^3^ | 145.77(4) | Br3 Hg1 Br5 | 103.61(4) |
| Br7^1^ Rb2 Br3 | 131.59(4) | Br7^1^ Rb2 Br5^3^ | 114.15(4) |
| Br2 Hg1 Br1 | 109.18(4) | Br3^3^ Rb2 Br7^9^ | 90.25(3) |
| Br3^3^ Rb2 Br5^3^ | 68.38(3) | Br3 Rb2 Br7^9^ | 136.48(4) |
| Br3 Rb2 Br5^3^ | 78.15(3) | Br3^3^ Rb2 Br3 | 82.63(4) |
| Br2 Hg1 Br3 | 128.61(5) | Br4 Hg2 Br5 | 98.70(4) |
| Br2 Hg1 Br5 | 107.74(5) | Br7 Hg2 Br5 | 91.61(3) |
| Br4 Hg2 Br7 | 122.14(4) | Br6 Hg2 Br4 | 116.57(4) |
| Br6 Hg2 Br5 | 100.80(4) | Br6 Hg2 Br7 | 116.91(4) |
| Br4^4^ Rb1 Br1^2^ | 136.44(5) | Br5^1^ Rb2Br1^3^ | 120.67(5) |
| Br4^4^ Rb1 Br3^4^ | 67.48(3) | Br5^1^ Rb2 Br7^1^ | 69.28(3) |
| Br4^4^ Rb1 Br6^5^ | 68.78(3) | Br5^3^ Rb2 Br7^9^ | 138.03(4) |
| Br4^4^ Rb1 Br5 | 79.09(3) | Br5^1^ Rb2 Br7^9^ | 129.73(4) |
| Br4^4^ Rb1 Br2^4^ | 79.19(3) | Br5^1^ Rb2 Br3^3^ | 139.99(4) |
| Br4^4^ Rb1 Br2^2^ | 129.27(4) | Br5^1^ Rb2 Br3 | 67.51(3) |
| Br4^4^ Rb1 Br2 | 138.97(4) | Hg2^1^ Rb3 Br1^11^ | 51.95(2) |
| Br1^2^ Rb1 Br3^4^ | 139.47(4) | Br4 Rb3 Br1^11^ | 131.94(4) |
| Br1^2^ Rb1 Br6^5^ | 67.92(3) | Br4 Rb3 Br1^12^ | 76.58(3) |
| Br1^2^ Rb1 Br5 | 139.34(4) | Br4 Rb3 Br7^1^ | 85.79(3) |
| Br1^2^ Rb1 Br2 | 72.90(3) | Br4 Rb3 Br7^9^ | 72.70(3) |
| Br1^2^ Rb1 Br2^2^ | 72.53(3) | Br4 Rb3 Br3^11^ | 161.59(5) |
| Br1^2^ Rb1 Br2^4^ | 80.65(3) | Br3^4^ Rb1 Br5 | 61.98(3) |
| Br3^4^ Rb1 Br2^4^ | 72.54(3) | Br3^4^ Rb1 Br2 | 111.13(4) |
| Br2 Rb1 Br2^4^ | 141.29(5) | Br4 Rb3 Br6^1^ | 73.90(3) |
| Br2^2^ Rb1 Br2^4^ | 64.31(4) | Br4 Rb3 Br2^10^ | 112.30(4) |
| Br2^2^ Rb1 Br2 | 80.84(4) | Br6^1^ Rb3 Br1^12^ | 128.50(4) |
| Br6 Rb1 Br4^4^ | 73.24(3) | Br6^1^ Rb3 Br1^11^ | 63.43(3) |
| Br6 Rb1 Br1^2^ | 97.62(4) | Br3^11^ Rb3 Br1^11^ | 64.94(3) |
| Br2^2^ Rb1 Br6^5^ | 121.42(4) | Br1^12^ Rb3 Br1^11^ | 148.66(4) |
| Br2^2^ Rb1 Br5 | 101.51(4) | Br1^12^ Rb3 Br7^1^ | 141.37(4) |
| Br6^5^ Rb1 Br3^4^ | 125.06(4) | Br1^12^ Rb3 Br7^9^ | 75.49(3) |
| Br6 Rb1 Br3^4^ | 122.70(4) | Br3^11^ Rb3 Br6^1^ | 124.50(4) |
| Br6 Rb1 Br6^5^ | 72.00(3) | Br3^11^ Rb3 Br2^10^ | 75.31(4) |
| Br6 Rb1 Br5 | 71.09(3) | Br3^11^ Rb3 Br1^12^ | 89.40(4) |
| Br6^5^ Rb1 Br5 | 136.46(4) | Br3^11^ Rb3 Br7^1^ | 98.42(4) |
| Br6 Rb1 Br2^4^ | 137.44(4) | Br3^11^ Rb3 Br7^9^ | 92.45(4) |
| Br6^5^ Rb1 Br2^4^ | 68.03(3) | Br7^9^ Rb3 Br1^11^ | 120.86(4) |
| Br6 Rb1 Br2 | 74.94(4) | Br7^1^ Rb3 Br1^11^ | 64.43(3) |
| Br6^5^ Rb1 Br2 | 123.63(4) | Br7^1^ Rb3 Br7^9^ | 66.47(2) |
| Br6 Rb1 Br2^2^ | 155.65(5) | Br5 Rb1 Br2 | 66.46(3) |
| Br5 Rb1 Br2^4^ | 134.22(4) | Br2^2^ Rb1 Br3^4^ | 68.66(3) |
| Br4 Rb2 Br1^3^ | 139.59(4) | Br6^1^ Rb3 Br7^9^ | 131.25(4) |
| Br4 Rb2 Br7^9^ | 71.25(3) | Br6^1^ Rb3 Br7^1^ | 76.66(3) |
| Br4 Rb2 Br7^1^ | 85.64(3) | Br4 Rb2 Br5^1^ | 86.21(4) |
| Br4 Rb2 Br3^3^ | 109.18(4) | Br1^3^ Rb2 Br7^9^ | 68.34(3) |
| Br4 Rb2 Br3 | 70.87(3) | Br1^3^ Rb2 Br7^1^ | 78.06(3) |
| Br4 Rb2 Br5^3^ | 148.91(4) | Br1^3^ Rb2 Br3^3^ | 70.75(3) |
| Br2^10^ Rb3 Br7^1^ | 141.22(5) | Br2^10^ Rb3 Br1^12^ | 77.37(4) |
| Br2^10^ Rb3 Br7^9^ | 150.27(5) | Br2^10^ Rb3 Br1^11^ | 78.80(4) |
| Br1^3^ Rb2 Br3 | 144.50(4) | Br2^10^ Rb3 Br6^1^ | 76.19(4) |

^1^+X, -1+Y, +Z; ^2^1-X, 2-Y, 1-Z; ^3^2-X, 1-Y, 1-Z; ^4^+X, 1+Y, +Z; ^5^1-X, 1/2+Y, 1/2-Z; ^6^+X, 3/2-Y, 1/2+Z; ^7^+X, 1/2-Y, 1/2+Z; ^8^2-X, 1/2+Y, 1/2-Z; ^9^2-X, -1/2+Y, 1/2-Z; ^10^1-X, -1/2+Y, 1/2-Z; ^11^+X, 1/2-Y, -1/2+Z; ^12^+X, 3/2-Y, -1/2+Z

**Table S22.** Atomic coordinates (× 10^4^), and equivalent isotropic displacement parameters (*U*_eq_^a^, Å^2^ × 10^3^) for Rb_3_Hg_2_I_7_. *U*_eq_ is defined as one third of the trace of the orthogonalized *U*_ij_ tensor.

| Atom | x | y | z | *U*_eq_^a^*/*Å^2^ |
| --- | --- | --- | --- | --- |
| Hg1 | 7276.3(4) | 7873.2(6) | 6963.7(3) | 59.31(14) |
| Hg2 | 7928.1(4) | 6868.8(6) | 4969.0(2) | 50.99(12) |
| I1 | 5431.9(6) | 9190.0(9) | 7263.1(4) | 48.75(18) |
| I2 | 6629.8(7) | 9014.5(9) | 5590.2(3) | 54.2(2) |
| I3 | 7760.7(6) | 8300.1(10) | 3777.6(3) | 52.89(19) |
| I4 | 9951.9(7) | 7134.5(13) | 5636.5(4) | 69.2(2) |
| I5 | 6771.3(7) | 3830.9(9) | 4790.4(4) | 52.38(19) |
| I6 | 7467.9(6) | 4379.3(8) | 6848.2(3) | 47.20(18) |
| I7 | 9151.9(6) | 9543.9(9) | 7369.4(4) | 52.07(19) |
| Rb1 | 5001.1(9) | 2620.7(16） | 6010.9(6) | 60.9(3) |
| Rb2 | 7421.5(11) | 2018.0(17) | 8267.4(5) | 63.3(3) |
| Rb3 | 9280.2(11) | 2111.6(18) | 5976.1(7) | 71.9(4) |

^a^*U*_eq_ is defined as one third of the trace of the orthogonalized *U*_ij_ tensor

**Table S23.** Anisotropic displacement parameters (Å^2^ × 10^3^) for Rb_3_Hg_2_I_7_. The anisotropic displacement factor exponent takes the form: -2π^2^[h^2^a^*2^U_11_+2hka^*^b^*^U_12_+…].

| Atom | U_11_ | U_22_ | U_33_ | U_23_ | U_13_ | U_12_ |
| --- | --- | --- | --- | --- | --- | --- |
| Hg1 | 46.8(3) | 44.7(2) | 86.4(4) | -9.6(2) | 13.0(3) | 1.0(2) |
| Hg2 | 45.2(3) | 60.1(3) | 44.4(2) | 1.6(2) | 0.9(2) | -3.7(2) |
| I1 | 41.5(4) | 48.2(4) | 58.8(5) | 4.1(3) | 15.4(3) | 4.1(3) |
| I2 | 67.4(5) | 52.6(4) | 43.4(4) | 2.5(3) | 12.5(4) | 22.8(4) |
| I3 | 51.5(4) | 67.6(5) | 39.3(4) | 10.6(3) | 8.5(3) | -1.6(4) |
| I4 | 40.6(4) | 98.0(7) | 62.1(5) | -11.8(5) | -6.8(4) | 7.6(4) |
| I5 | 64.6(5) | 43.1(4) | 52.0(4) | 1.4(3) | 17.6(4) | -7.1(3) |
| I6 | 52.7(4) | 36.7(3) | 53.2(4) | -1.1(3) | 12.9(4) | 2.5(3) |
| I7 | 43.9(4) | 51.8(4) | 58.1(5) | 2.4(3) | 4.2(3) | -6.0(3) |
| Rb1 | 41.7(6) | 70.8(8) | 67.3(8) | 6.6(6) | 3.5(6) | 8.1(5) |
| Rb2 | 73.0(9) | 71.0(8) | 42.9(6) | -2.1(6) | 4.2(6) | -1.0(6) |
| Rb3 | 61.2(8) | 80.0(9) | 82.3(9) | 17.5(7) | 33.0(7) | 15.5(7) |

**Table S24.** Bond lengths for Rb_3_Hg_2_I_7_.

| Atom | Length/ Å | Atom | Length/ Å |
| --- | --- | --- | --- |
| Hg2 I5 | 2.7602(9) | I7 Rb3^2^ | 3.7224(15) |
| Hg2 I2 | 2.8669(8) | I5 Rb1 | 3.9316(14) |
| Hg2 I3 | 2.8045(8) | I5 Rb1^1^ | 3.7611(14) |
| Hg2 I4 | 2.7054(9) | I5 Rb2^5^ | 3.6513(14) |
| I7 Rb2^2^ | 3.7148(15) | I5 Rb3 | 3.9280(17) |
| Hg1 I1 | 2.7517(8) | I2 Rb1^1^ | 3.9167(15) |
| Hg1 I6 | 2.7316(8) | I2 Rb1^2^ | 3.6985(14) |
| Hg1 I7 | 2.7141(9) | I2 Rb3^2^ | 4.0857(17) |
| Hg1 I2 | 3.0880(9) | I3 Rb1^1^ | 3.6999(14) |
| I1 Rb1^3^ | 4.1103(15) | I3 Rb2^5^ | 4.2652(15) |
| I1 Rb1^2^ | 3.7796(14) | I3 Rb3^7^ | 3.7075(15) |
| I1 Rb2^3^ | 3.9593(16) | I4 Rb2^4^ | 3.7105(16) |
| I1 Rb2^2^ | 3.7202(15) | I4 Rb3 | 4.0804(17) |
| I6 Rb1 | 3.5760(15) | I4 Rb3^7^ | 3.8898(16) |
| I6 Rb2 | 3.6168(14) | I4 Rb3^2^ | 4.0466(17) |
| I6 Rb3 | 3.7110(14) | I3 Rb2^6^ | 3.7920(15) |
| I7 Rb3^4^ | 4.1752(18) |  |  |

^1^1-X, 1-Y, 1-Z; ^2^+X, 1+Y, +Z; ^3^1-X, 1/2+Y, 3/2-Z; ^4^2-X, 1/2+Y, 3/2-Z; ^5^+X, 1/2-Y, -1/2+Z; ^6^+X, 3/2-Y, -1/2+Z; ^7^2-X, 1-Y, 1-Z

**Table S25.** Bond angles for Rb_3_Hg_2_I_7_.

| Atom | Angle/° | Atom | | | Angle/° |
| --- | --- | --- | --- | --- | --- |
| I5 Hg2 I2 | 102.73(3) | I1^8^ Rb2 I3^11^ | | | 121.75(4) |
| I5 Hg2 I3 | 105.31(3) | I7^9^ Rb2 I1^8^ | | | 132.00(4) |
| I3 Hg2 I2 | 105.52(3) | I3^1^ Rb1 I1^8^ | | | 68.87(3) |
| I4 Hg2 I5 | 124.39(3) | I3^1^ Rb1 I1^9^ | | | 77.25(3) |
| I4 Hg2 I2 | 105.93(3) | I3^1^ Rb1 I5 | | | 144.61(4) |
| I4 Hg2 I3 | 111.28(3) | I3^1^ Rb1 I5^1^ | | | 72.72(3) |
| I1 Hg1 I2 | 92.88(3) | I3^1^ Rb1 I2^1^ | | | 72.65(3) |
| I6 Hg1 I1 | 118.98(3) | I6 Rb2 I7^9^ | | | 73.00(3) |
| I6 Hg1 I2 | 101.80(3) | I6 Rb2 I5^11^ | | | 157.12(4) |
| I7 Hg1 I1 | 118.13(3) | I6 Rb2 I3^10^ | | | 75.54(3) |
| I7 Hg1 I6 | 114.58(3) | I6 Rb2 I3^11^ | | | 134.03(4) |
| I7 Hg1 I2 | 104.54(3) | I6 Rb2 I4^12^ | | | 112.28(4) |
| I5^11^ Rb2 I1^8^ | 89.17(3) | I6 Rb2 Rb1 | | | 42.19(2) |
| I5^11^ Rb2 I1^9^ | 100.64(4) | I7^9^ Rb2 I1^9^ | | | 78.19(3) |
| I5^11^ Rb2 I7^9^ | 129.79(4) | I7^9^ Rb2 I3^11^ | | | 66.26(3) |
| I6 Rb2 I1^8^ | 72.67(3) | I7^9^ Rb2 I3^10^ | | | 126.84(4) |
| I6 Rb2 I1^9^ | 85.02(3) | I5^11^ Rb2 I3^11^ | | | 67.43(2) |
| I2^9^ Rb1 I1^9^ | 69.04(3) | I5^11^ Rb2 I3^10^ | | | 86.31(3) |
| I2^9^ Rb1 I1^8^ | 129.43(4) | I5^11^ Rb2 I4^12^ | | | 75.51(3) |
| I2^1^ Rb1 I1^8^ | 140.91(4) | I3^10^ Rb2 I1^8^ | | | 74.05(3) |
| I2^9^ Rb1 I5 | 65.61(3) | I3^10^ Rb2 I3^11^ | | | 147.71(4) |
| I2^9^ Rb1 I5^1^ | 138.44(4) | I4^12^ Rb2 I1^8^ | | | 146.66(4) |
| I2^1^ Rb1 I5 | 74.99(3) | I4^12^ Rb2 I1^9^ | | | 144.84(4) |
| I2^9^ Rb1 I2^1^ | 75.91(3) | I4^12^ Rb2 I7^9^ | | | 78.26(3) |
| I2^9^ Rb1 I3^1^ | 118.51(4) | I4^12^ Rb2 I3^11^ | | | 79.76(3) |
| I1^9^ Rb2 I1^8^ | 66.38(2) | I4^12^ Rb2 I3^10^ | | | 75.55(3) |
| I1^9^ Rb2 I3^10^ | 139.58(4) | I6 Rb3 I7^12^ | | | 65.64(3) |
| I1^9^ Rb2 I3^11^ | 66.98(3) | I6 Rb3 I7^9^ | | | 71.85(3) |
| I1^9^ Rb1 I1^8^ | 64.31(2) | I6 Rb3 I5 | | | 71.01(3) |
| I1^9^ Rb1 I5 | 129.82(3) | I6 Rb3 I2^9^ | | | 79.27(3) |
| I1^9^ Rb1 I2^1^ | 113.48(4) | I6 Rb3 I4^9^ | | | 136.04(4) |
| I6 Rb1 I1^9^ | 84.72(3) | I6 Rb3 I4 | | | 79.43(3) |
| I6 Rb1 I1^8^ | 71.24(3) | I6 Rb3 I4^7^ | | | 133.53(4) |
| I6 Rb1 I5 | 72.34(3) | I7^9^ Rb3 I7^12^ | | | 67.61(3) |
| I6 Rb1 I5^1^ | 110.23(4) | I7^9^ Rb3 I5 | | | 124.76(4) |
| I6 Rb1 I2^9^ | 86.43(3) | I7^9^ Rb3 I2^9^ | | | 71.92(3) |
| I6 Rb1 I2^1^ | 146.99(4) | I7^9^ Rb3 I4^9^ | | | 71.99(3) |
| I6 Rb1 I3^1^ | 140.09(4) | I7^9^ Rb3 I4^7^ | | | 154.06(4) |
| I5 Rb1 I1^8^ | 138.88(4) | I3^7^ Rb3 I5 | | | 142.55(4) |
| I5^1^ Rb1 I1^9^ | 147.12(4) | I3^7^ Rb3 I2^9^ | | | 138.72(4) |
| I5^1^ Rb1 I1^8^ | 92.13(3) | I3^7^ Rb3 I4 | | | 82.28(3) |
| I5^1^ Rb1 I5 | 83.06(3) | I3^7^ Rb3 I4^7^ | | | 73.52(3) |
| I5^1^ Rb1 I2^1^ | 69.83(3) | I3^7^ Rb3 I4^9^ | | | 72.55(3) |
| I7^9^ Rb3 I4 | 134.94(5) | I4^7^ Rb3 I7^12^ | | | 123.02(4) |
| I5 Rb3 I7^12^ | 126.94(4) | I4^9^ Rb3 I7^12^ | | | 120.25(4) |
| I5 Rb3 I2^9^ | 62.15(3) | I4 Rb3 I7^12^ | | | 69.15(3) |
| I5 Rb3 I4^9^ | 111.98(4) | I4^7^ Rb3 I5 | | | 70.42(3) |
| I5 Rb3 I4 | 74.24(3) | I4 Rb3 I2^9^ | | | 135.65(4) |
| I2^9^ Rb3 I7^12^ | 132.60(3) | I4^7^ Rb3 I2^9^ | | | 104.14(4) |
| I3^7^ Rb3 I6 | 132.90(5) | I4^9^ Rb3 I2^9^ | | | 66.33(3) |
| I3^7^ Rb3 I7^9^ | 92.44(4) | I4^7^ Rb3 I4 | | | 65.96(3) |
| I4^9^ Rb3 I4 | 144.52(4) | I4^7^ Rb3 I4^9^ | | | 82.88(3) |
| I3^7^ Rb3 I7^12^ | 67.31(3) |  |  |  |  |

^1^1-X, 1-Y, 1-Z; ^2^+X, 1+Y, +Z; ^3^1-X, 1/2+Y, 3/2-Z; ^4^2-X, 1/2+Y, 3/2-Z; ^5^+X, 1/2-Y, -1/2+Z; ^6^+X, 3/2-Y, -1/2+Z; ^7^2-X, 1-Y, 1-Z; ^8^1-X, -1/2+Y, 3/2-Z; ^9^+X, -1+Y, +Z; ^10^+X, 3/2-Y, 1/2+Z; ^11^+X, 1/2-Y, 1/2+Z; ^12^2-X, -1/2+Y, 3/2-Z


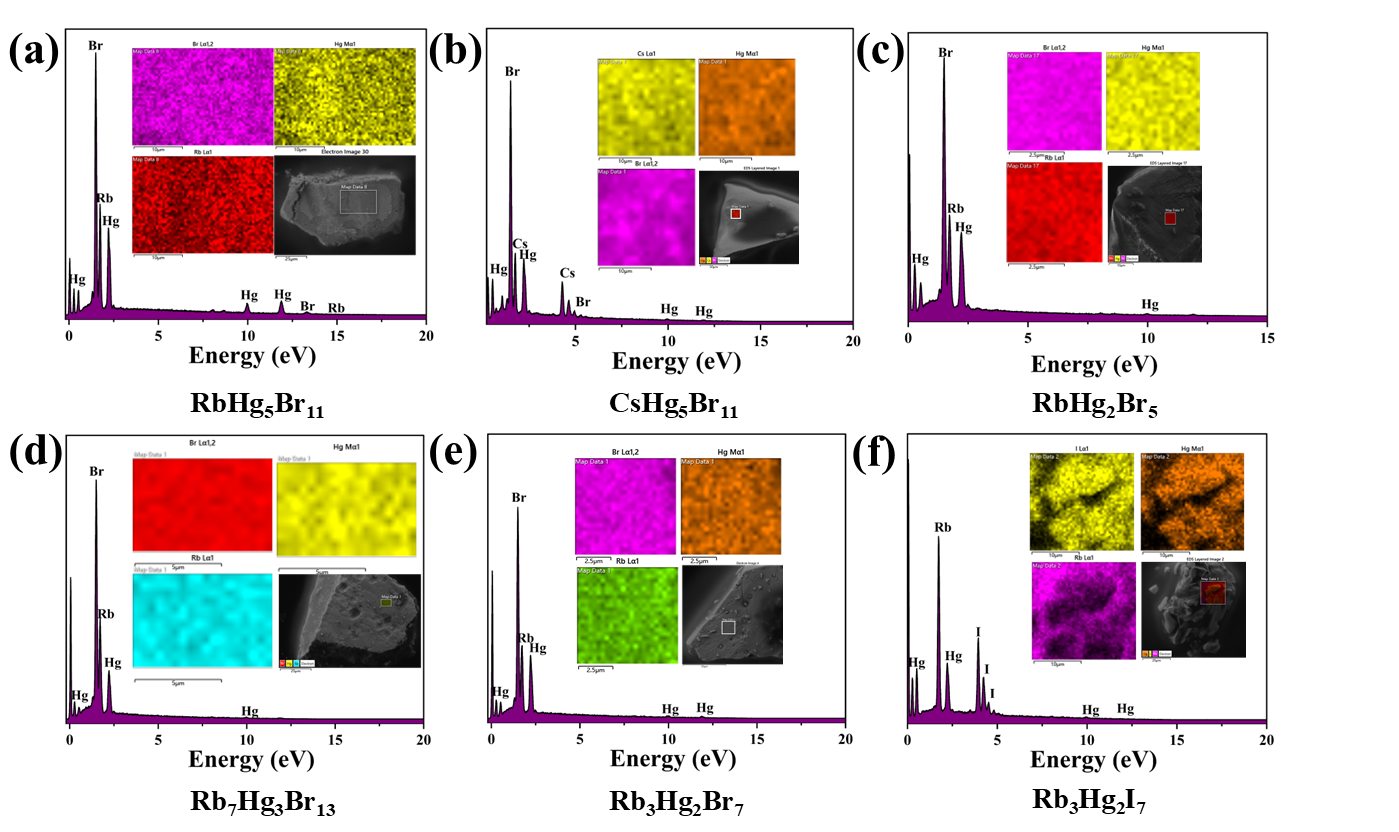


Figure S1. The results of element analyses for RbHg_5_Br_11_, CsHg_5_Br_11_, RbHg_2_Br_5_, Rb_7_Hg_3_Br_13_, Rb_3_Hg_2_Br_7_ and Rb_3_Hg_2_I_7_.


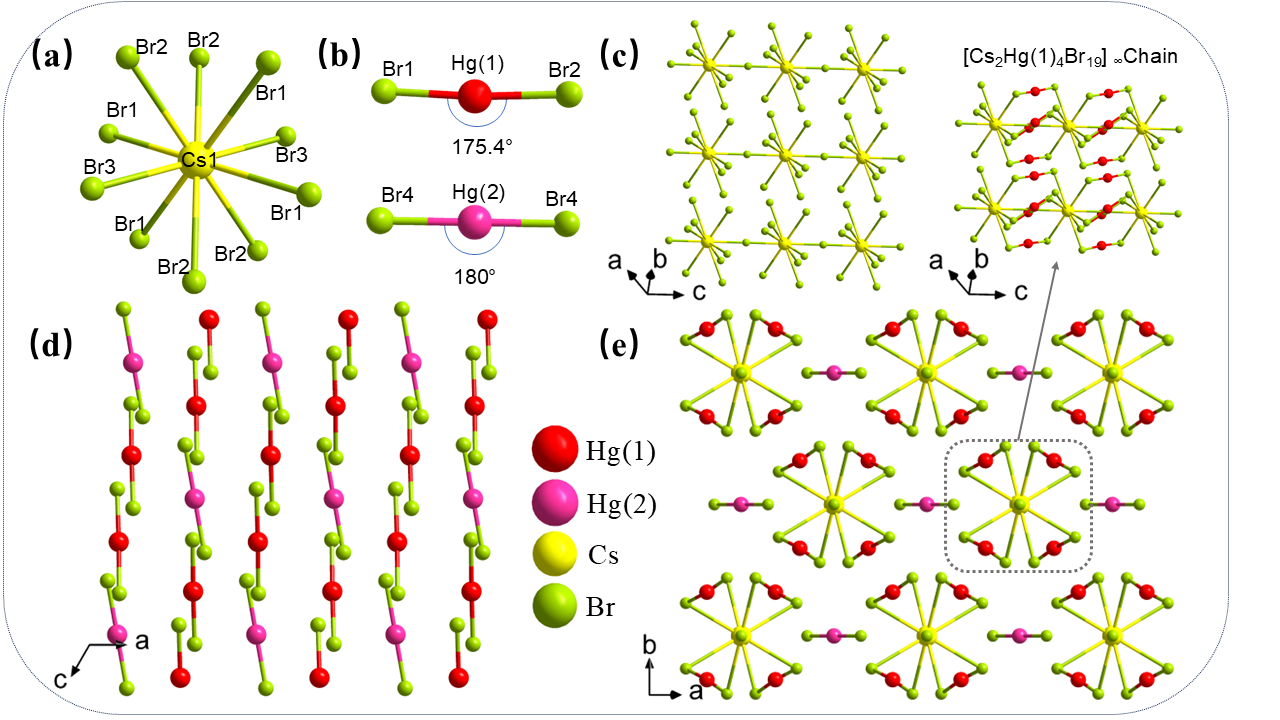


Figure S2. Crystal structure of CsHg_5_Br_11_. (a-b) Coordination modes of Cs and Hg; (c) The formed Cs-Br framework; (d) The arrangement of the isolated [Hg(1)Br_2_] and [Hg(2)Br_2_] units; (e) The resulting 3D crystal structure.





**Figure S3.** The Raman spectrum of RbHg_5_Br_11_.


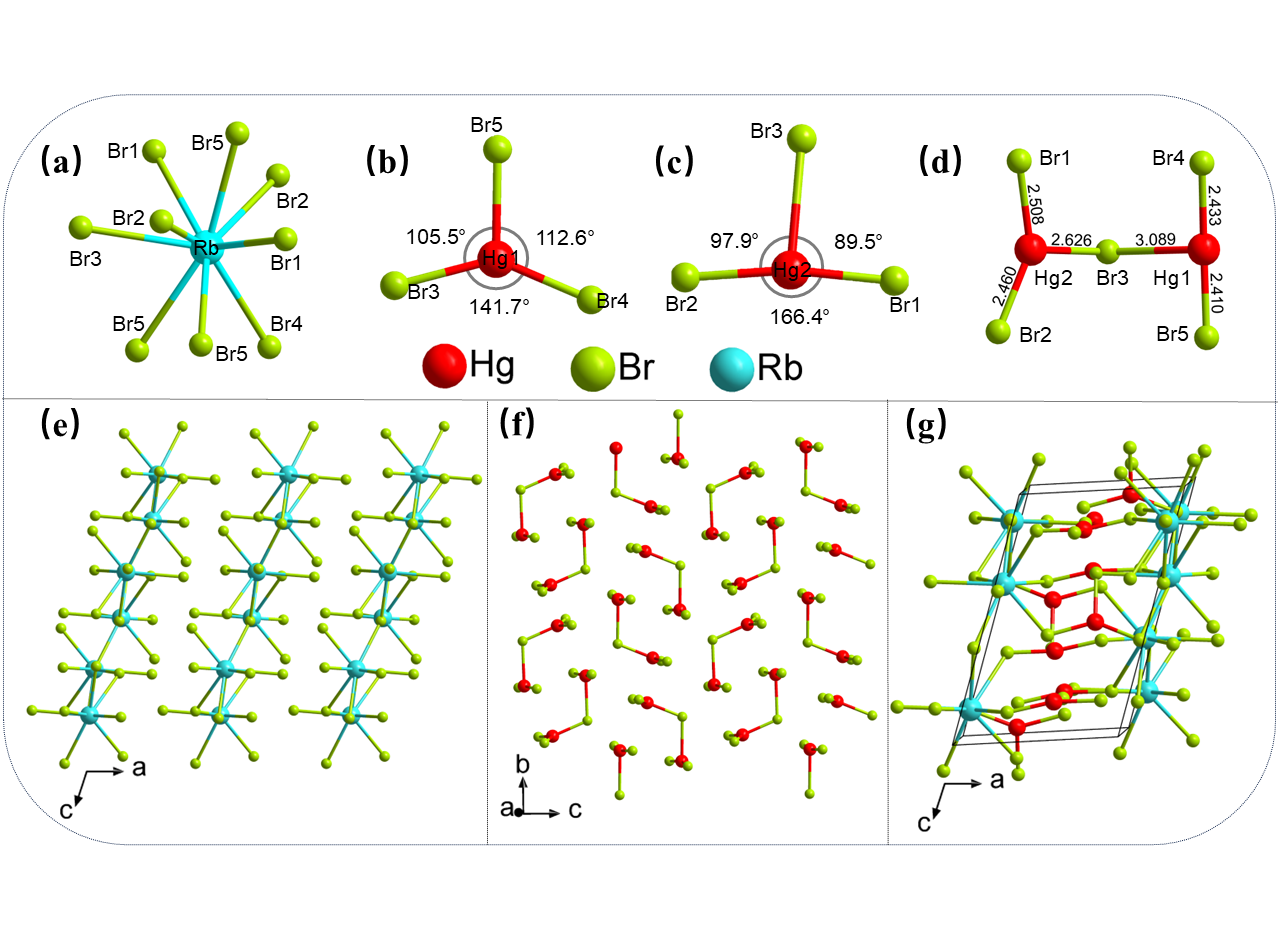


**Figure S4.** Crystal structure of RbHg_2_Br_5_. (a-d) Coordination modes of Rb and Hg; (e) The formed Rb-Br framework; (f) The arrangement of the isolated [Hg_2_Br_5_] unit; (g) The resulting 3D crystal structure.


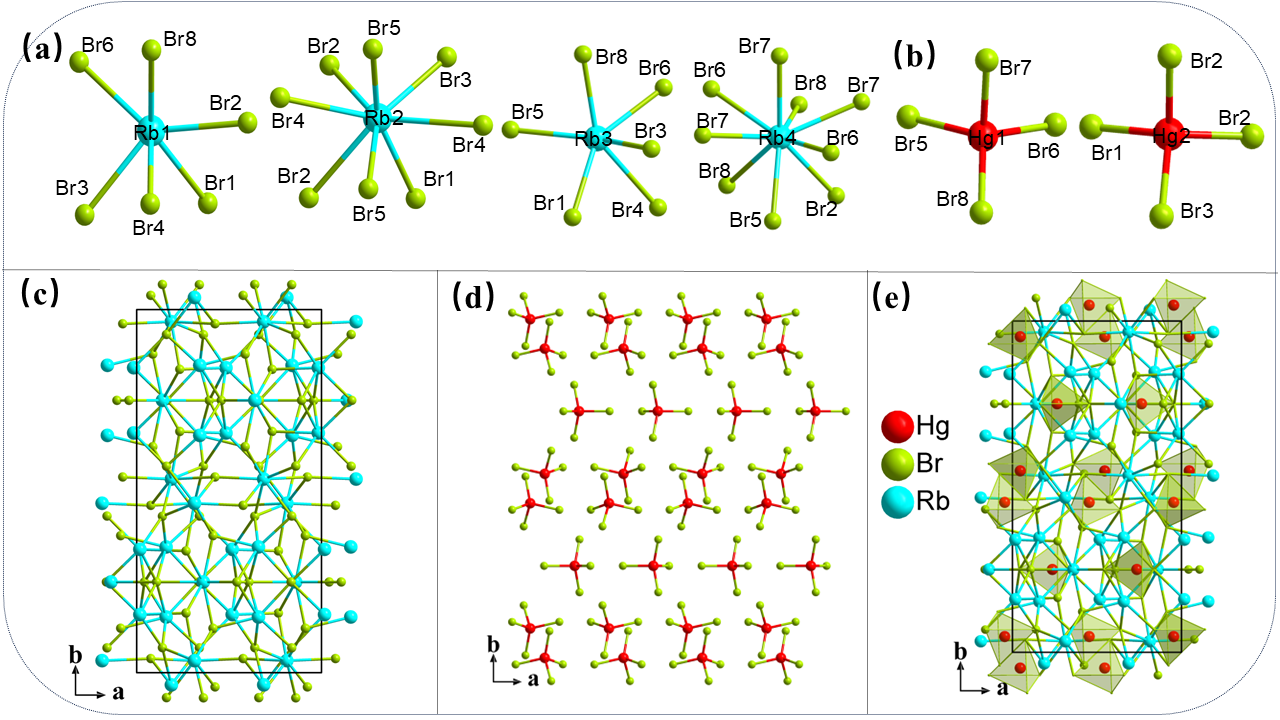


**Figure S5.** Crystal structure of Rb_7_Hg_3_Br_13_. (a-b) Coordination modes of Rb and Hg; (c) The formed Rb-Br framework; (d) The resulting [HgBr_4_] pseudo-layer; (e) The 3D crystal structure.


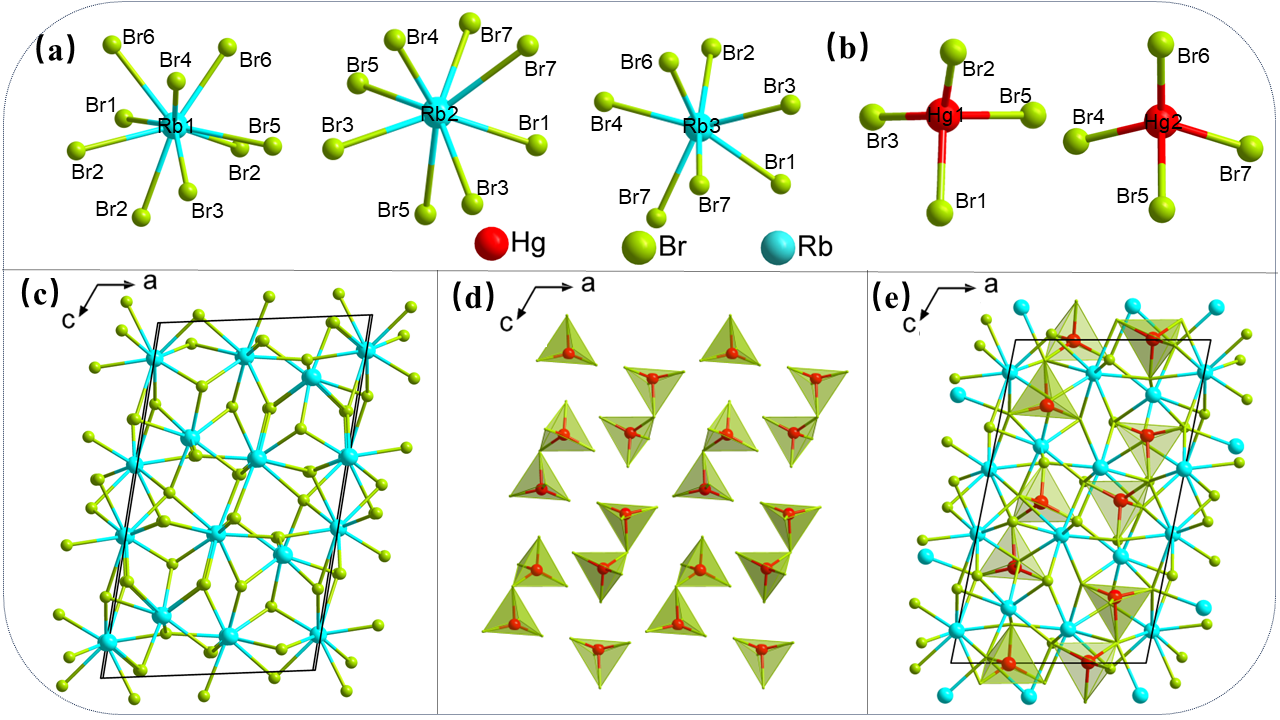


**Figure S6.** Crystal structure of Rb_3_Hg_2_Br_7_. (a-b) Coordination modes of Rb and Hg; (c) The formed Rb-Br framework; (d) The arrangement of isolated [Hg_2_Br_7_] unit; (e) The resulted 3D crystal structure.


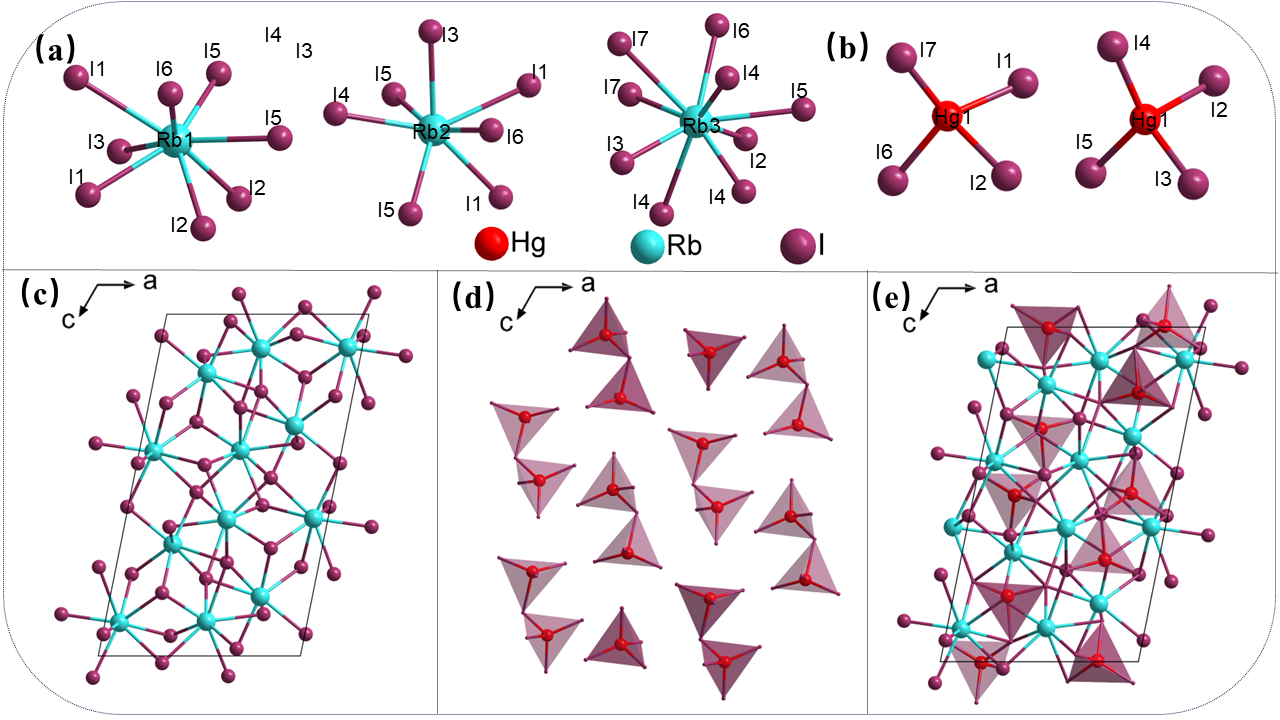


**Figure S7.** Crystal structure of Rb_3_Hg_2_I_7_. (a, b) Coordination modes of Rb and Hg; (c) The formed Rb-I framework; (d) The arrangement of isolated [Hg_2_I_7_] unit; (e) The resulted 3D crystal structure.


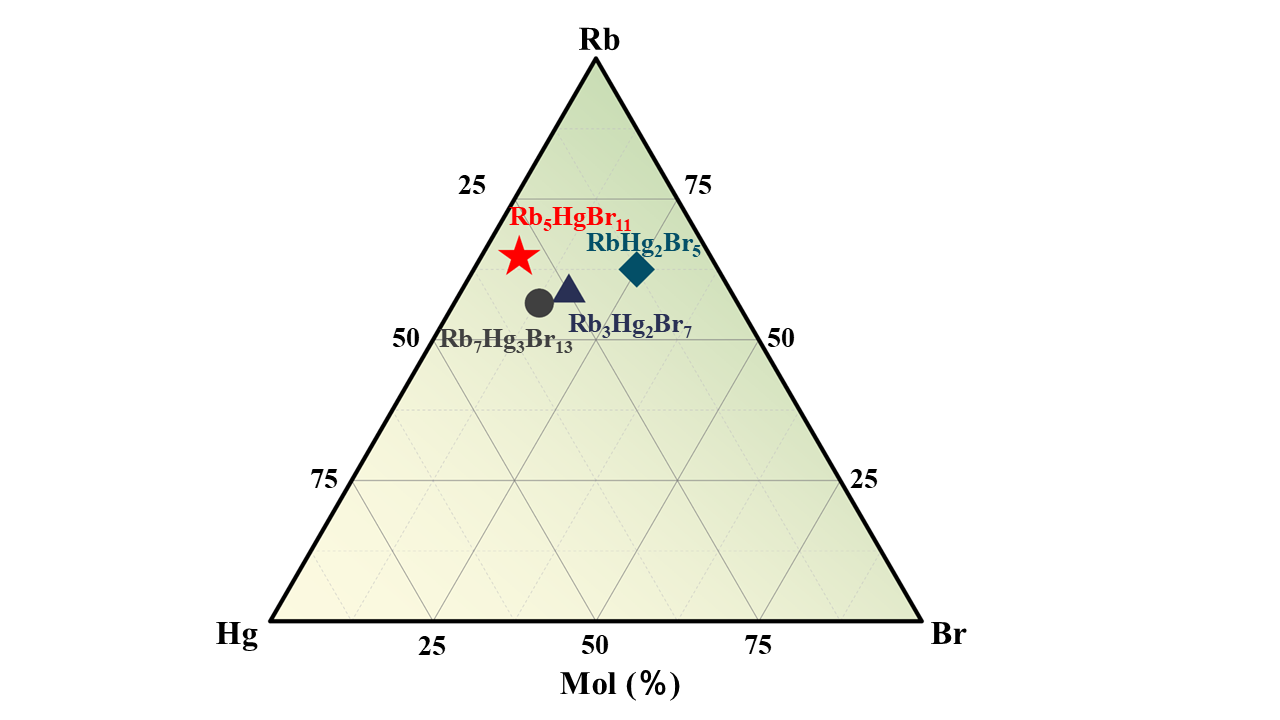


Figure S8 Ternary diagram for the Rb-Hg-Br system.





**Figure S9.** The calculated birefringence for the series of compounds (CsHg_5_Br_11_, RbHg_2_Br_5_, Rb_7_Hg_3_Br_13_, Rb_3_Hg_2_Br_7_ and Rb_3_Hg_2_I_7_).


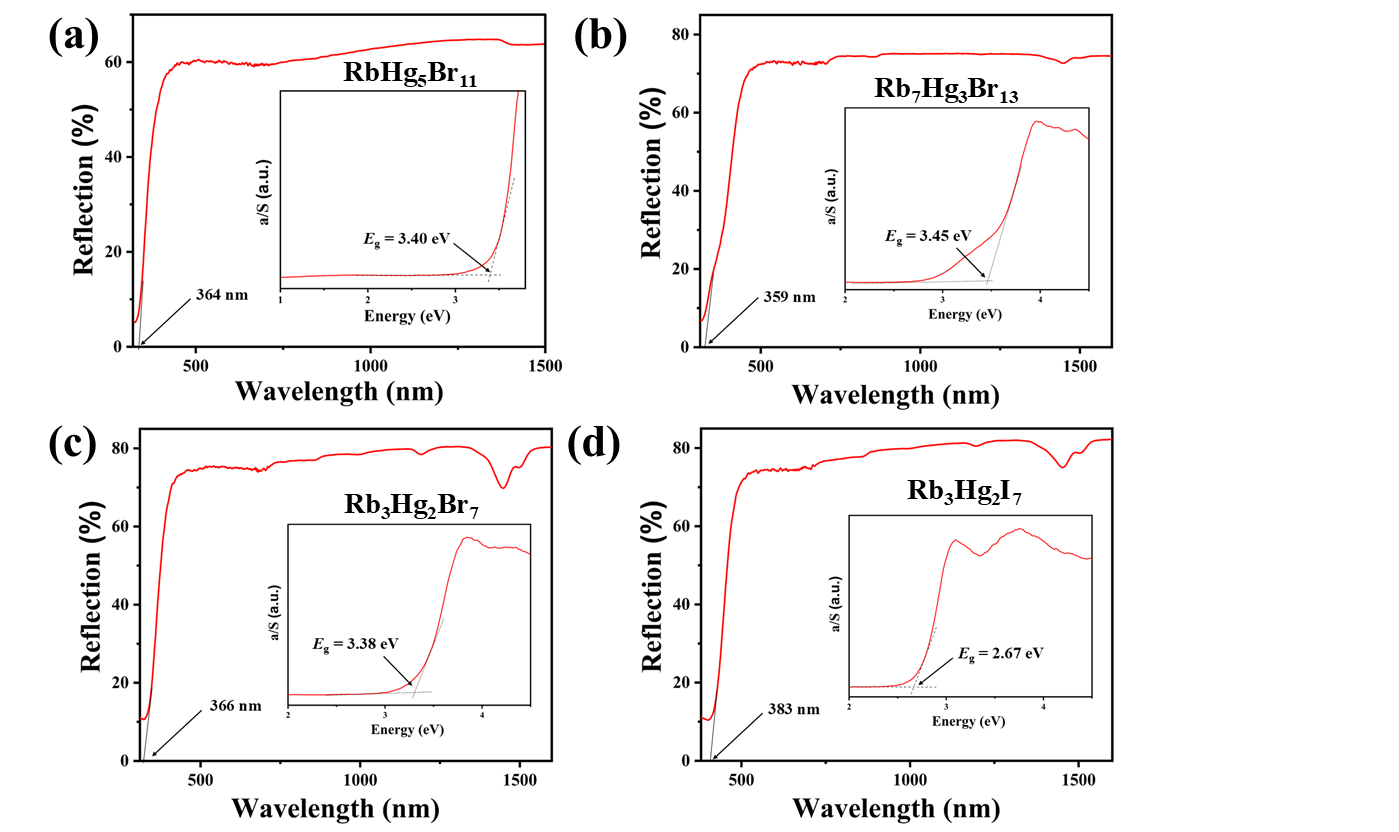


**Figure S10.** The experimental band gaps of RbHg_5_Br_11_, Rb_7_Hg_3_Br_13_, Rb_3_Hg_2_Br_7_ and Rb_3_Hg_2_I_7_.


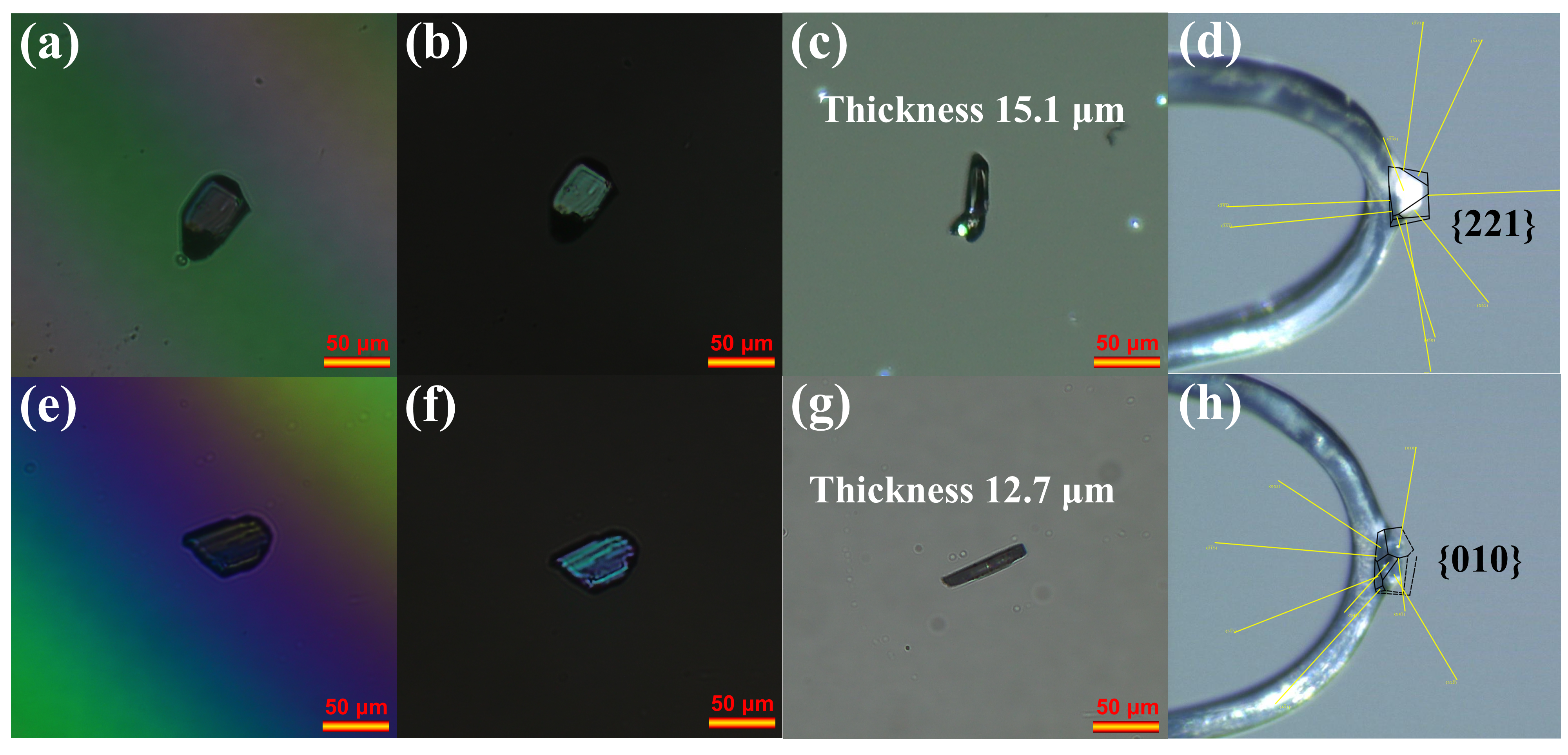


**Figure S11.** a, e) The rotation of compensatory; b, f) Photographs of CsHg_5_Br_11_ and RbHg_2_Br_5_ crystal; c, g) The thicknesses of CsHg_5_Br_11_ and RbHg_2_Br_5_ for the RID measurements; d, h) The crystal orientations of CsHg_5_Br_11_ and RbHg_2_Br_5_ single crystals indexed by single-crystal XRDs.





**Figure S12.** The powder XRD patterns for RbHg_5_Br_11_ before and after exposure in air for six months, the theoretical results utilized as the reference.


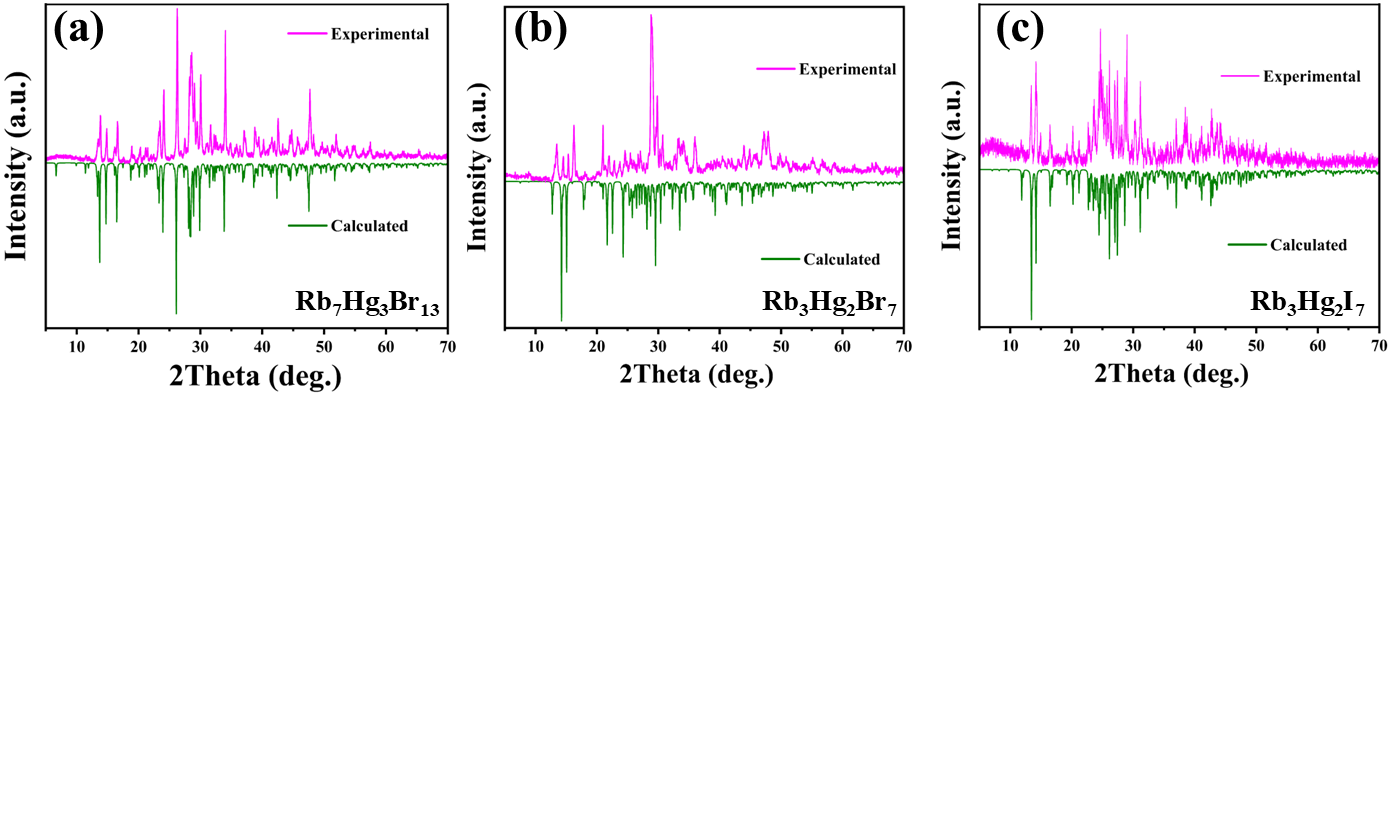


**Figure S13.** The powder XRD patterns of Rb_7_Hg_3_Br_13_ (a), Rb_3_Hg_2_Br_7_ (b) and Rb_3_Hg_2_I_7_ (c) polycrystalline pure phase samples.


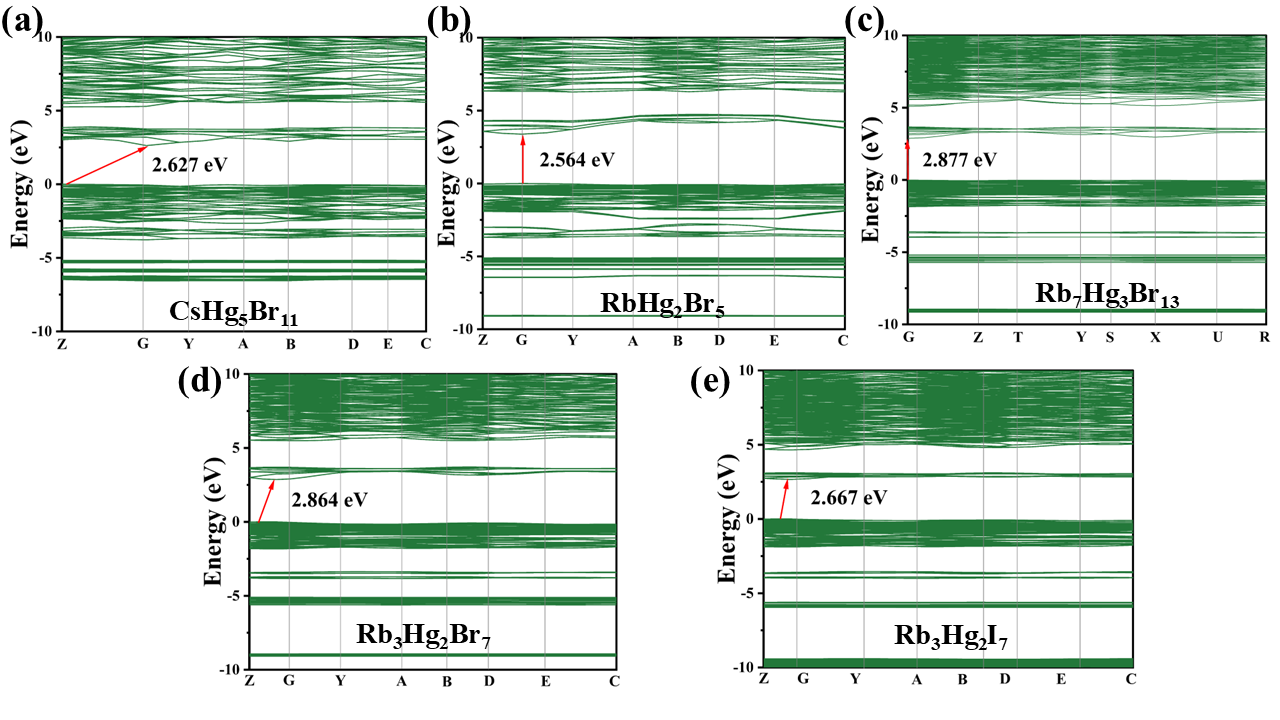


**Figure S14.** The band structures of CsHg_5_Br_11_ (a), RbHg_2_Br_5_ (b), Rb_7_Hg_3_Br_13_ (c), Rb_3_Hg_2_Br_7_ (d) and Rb_3_Hg_2_I_7_ (e).


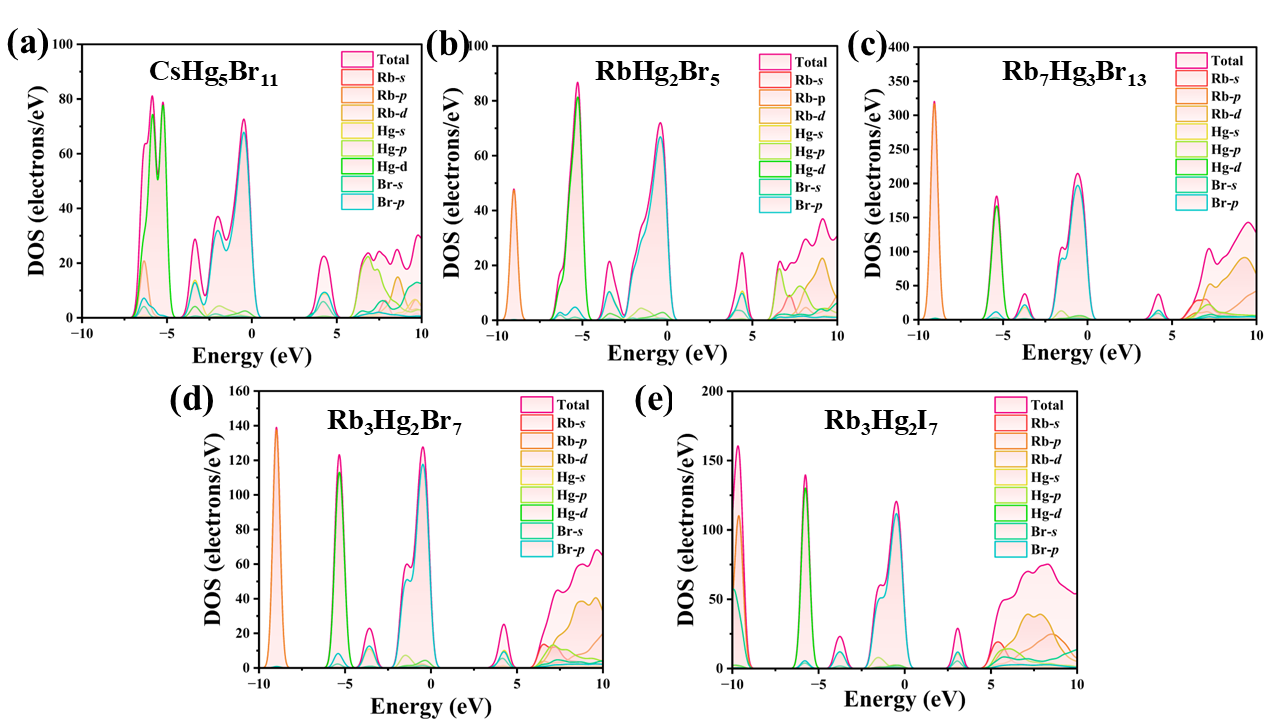


**Figure S15.** The total/partial density of states (T/PDOS) of CsHg_5_Br_11_ (a), RbHg_2_Br_5_ (b), Rb_7_Hg_3_Br_13_ (c), Rb_3_Hg_2_Br_7_ (d) and Rb_3_Hg_2_I_7_ (e).

**References**

1. H. T. Luo, T. Tkaczyk, E. L. Dereniak, K. Oka, R. Sampson, *Opt. Lett.* **2006**, *31*, 616.
2. D. E. Zelmon, D. L. Small, D. Jundt, *J. Opt. Soc. Am. B.* **1997**, *14*, 3319.
3. R. Devore, *J. Opt. Soc. Am.* **1950**, *40*, 266.
4. G. Ghosh, *Opt. Commun.* **1999**, *163*, 95.
5. D. M. Ceperley, B. J. Alder, *Phys. Rev. Lett.* **1980**, *45*, 566.
6. M. J. Dodge, *Appl. Opt.* **1984**, *23*, 1980.
7. J. Y. Guo, A. Tudi, S. J. Han, Z. H. Yang, S. L. Pan, *Angew. Chem. Int. Ed.* **2021**, *60*, 3540.
8. J. Réhault, R. Borrego-Varillas, A. Oriana, C. Manzoni, C. P. Hauri, J. Helbing, *G. Cerullo, Opt. Express*. **2017**, *25*, 4403.
9. L. Liu, R. Z. Li, L. Z. Zhang, P. Zhang, G. D. Zhang, S. Q. Xia, X. T. Tao, *J. Alloys Compd.* **2021**, *874*, 159943.
10. D. L. Porokhovnichenko, E. A. Dyakonov, J. Ryu, V. I. Balakshy, *Opt. Eng*. **2021**, *60*, 020501.
11. M. S. Zhang, W. D. Yao, S. M. Pei, B. W. Liu, X. M. Jiang, G. C. Guo, *Chem. Sci.* **2024**, *15*, 6891.
12. Y. L. Lv, R. L. Tang, C. Chen, L. Ma, W. L. Liu, S. P. Guo, *Adv.Opt. Mater.* **2025**, https://doi.org/10.1002/adom.202501474.
13. G. Zhang, Y. J. Li, K. Jiang, H. Y. Zeng, T. Liu, X. G. Chen, J. G. Qin, Z. S. Lin, P. Z. Fu, Y. C. Wu, C. T. Chen, *J. Am. Chem. Soc.* **2012**, *134*, 14818.
14. G. Zhang, J. G. Qin, T. Liu, T. X. Zhu, P. Z. Fu, Y. C. Wu, C. T. Chen, *Cryst. Growth Des.* **2008**, *8*, 2946.
15. Y. Y. Kong, H. S. Wang, W. Zhao, Q. Sun, J. J. Li, S. L. Pan, *Dalton Trans.* **2024**, *53*, 12090.
16. Q. Wu, Y. Huang, X. G. Meng, C. Zhong, X. G. Chen, J. G. Qin, *Dalton Trans.* **2014**, *43*, 8899.
17. Y. J. Li, Y. X. Ding, Y. M. Li, H. M. Liu, X. G. Meng, Y. Cong, J. Zhang, X. K. Li, X. G. Chen, J. G. Qin, *Crystals* **2017**, *7*, 148.
18. L. Huang, Q. Wang, F. F. He, X. Y. Liu, Z. W. Chen, W. He, X. Y. Luo, D. J. Gao, J. Bi, G. H. Zou, *J. Alloys Compd.* **2019**, *771*, 547.
19. Q. Wu, C. Yang, X. Liu, J. Ma, F. Liang, Y. S. Du, *Mater. Today Phys.* **2021**, *21*, 100569.
